# Reorganization of brain networks and its association with general cognitive performance over the adult lifespan

**DOI:** 10.1038/s41598-019-47922-x

**Published:** 2019-08-06

**Authors:** Epifanio Bagarinao, Hirohisa Watanabe, Satoshi Maesawa, Daisuke Mori, Kazuhiro Hara, Kazuya Kawabata, Noritaka Yoneyama, Reiko Ohdake, Kazunori Imai, Michihito Masuda, Takamasa Yokoi, Aya Ogura, Toshiaki Taoka, Shuji Koyama, Hiroki C. Tanabe, Masahisa Katsuno, Toshihiko Wakabayashi, Masafumi Kuzuya, Norio Ozaki, Minoru Hoshiyama, Haruo Isoda, Shinji Naganawa, Gen Sobue

**Affiliations:** 1 Brain and Mind Research Center, Nagoya University, Nagoya, Aichi Japan; 2 Department of Neurology, Nagoya University Graduate School of Medicine, Nagoya, Aichi Japan; 3 Department of Neurosurgery, Nagoya University Graduate School of Medicine, Nagoya, Aichi Japan; 4 Department of Radiology, Nagoya University Graduate School of Medicine, Nagoya, Aichi Japan; 5 Department of Cognitive and Psychological Sciences, Graduate School of Informatics, Nagoya University, Nagoya, Aichi Japan; 6 Department of Community Healthcare and Geriatrics, Nagoya University Graduate School of Medicine and Institutes of Innovation for Future Society, Nagoya University, Nagoya, Aichi Japan; 7 Department of Psychiatry, Nagoya University Graduate School of Medicine, Nagoya, Aichi Japan; 8 Department of Neurology, Fujita Health University School of Medicine, Toyoake, Aichi Japan

**Keywords:** Ageing, Cognitive ageing, Ageing

## Abstract

Healthy aging is associated with structural and functional changes in the brain even in individuals who are free of neurodegenerative diseases. Using resting state functional magnetic resonance imaging data from a carefully selected cohort of participants, we examined cross sectional changes in the functional organization of several large-scale brain networks over the adult lifespan and its potential association with general cognitive performance. Converging results from multiple analyses at the voxel, node, and network levels showed widespread reorganization of functional brain networks with increasing age. Specifically, the primary processing (visual and sensorimotor) and visuospatial (dorsal attention) networks showed diminished network integrity, while the so-called core neurocognitive (executive control, salience, and default mode) and basal ganglia networks exhibited relatively preserved between-network connections. The visuospatial and precuneus networks also showed significantly more widespread increased connectivity with other networks. Graph analysis suggested that this reorganization progressed towards a more integrated network topology. General cognitive performance, assessed by Addenbrooke’s Cognitive Examination-Revised total score, was positively correlated with between-network connectivity among the core neurocognitive and basal ganglia networks and the integrity of the primary processing and visuospatial networks. Mediation analyses further indicated that the observed association between aging and relative decline in cognitive performance could be mediated by changes in relevant functional connectivity measures. Overall, these findings provided further evidence supporting widespread age-related brain network reorganization and its potential association with general cognitive performance during healthy aging.

## Introduction

The structural and functional changes the brain undergoes due to the aging process have been studied for many years. Although the rate of anatomical change varies from one brain region to another, the aging brain is typically characterized by an increase in gray matter atrophy^1–4^ as well as an overall decline of white matter volume at old age^1,5^. Significant age-related differences in brain activation pattern can also be observed in the elderly adult compared to young adults^6,7^. Specifically, the elderly’s brain tends to exhibit increase in brain activity, often seen in the frontal regions^8,9^, and to recruit more regions of both hemispheres leading to a decrease in functional lateralization^10^. Aging is also associated with behavioral changes, which could manifest as a reduction in the ability to perform tasks involving high level “executive” functions such as episodic memory^11,12^, working memory^11,13^, attention^14^, and task switching^15^. Nevertheless, some aspects of cognition such as semantic memory (e.g. maintenance of general knowledge and vocabulary) and emotion regulation remained intact^16–18^. Identifying the relationship among structural, functional, and behavioral changes associated with the aging process still remains a challenge.

Resting state functional magnetic resonance imaging (rsfMRI), which measures the intrinsic activity of the brain when participants are not engaged in any particular activity (task-free state), has enabled the investigation of the brain’s intrinsic functional organization. Using rsfMRI, large-scale resting state networks (RSNs) such as sensorimotor, default mode, salience, executive control, and attention networks, among others, have been identified^19–23^. Several studies have investigated the effects of aging in these networks across the lifespan^24–29^. A common finding was the observed decrease in connectivity within the default mode, salience, and executive control/attention networks with age^24–28,30–32^. Findings for other RSNs such as sensorimotor and subcortical networks were somewhat inconsistent with some showing increases^27,32^, no change^31^, or non-linear changes^28^ in connectivity with age. Other studies have used graph theory to identify the age-related changes in the brain’s network topology. In terms of network measures, modularity (a measure quantifying the degree to which a network could be subdivided into non-overlapping groups) and local efficiency (a measure characterizing how well a given neighborhood exchanged information when a node is removed)^31,32^ as well as global efficiency (a measure of network integration quantifying how well information is exchanged across the whole network)^33^ have been shown to decrease with age. Segregation in brain systems, which reflects dense within-system but parser between-system connectivity and quantified using network measures such as modularity and participation coefficient (a measure of the extent a given node connects to nodes in other systems), has also been shown to decrease across the adult lifespan^29^, although opposite findings have also been reported^34^. Overall, these studies have provided insights into how the brain’s functional organization is altered during healthy aging. However, little is known about the effects of age-related network reorganization on the observed individual differences in general cognitive performance with age.

**Figure 1:**
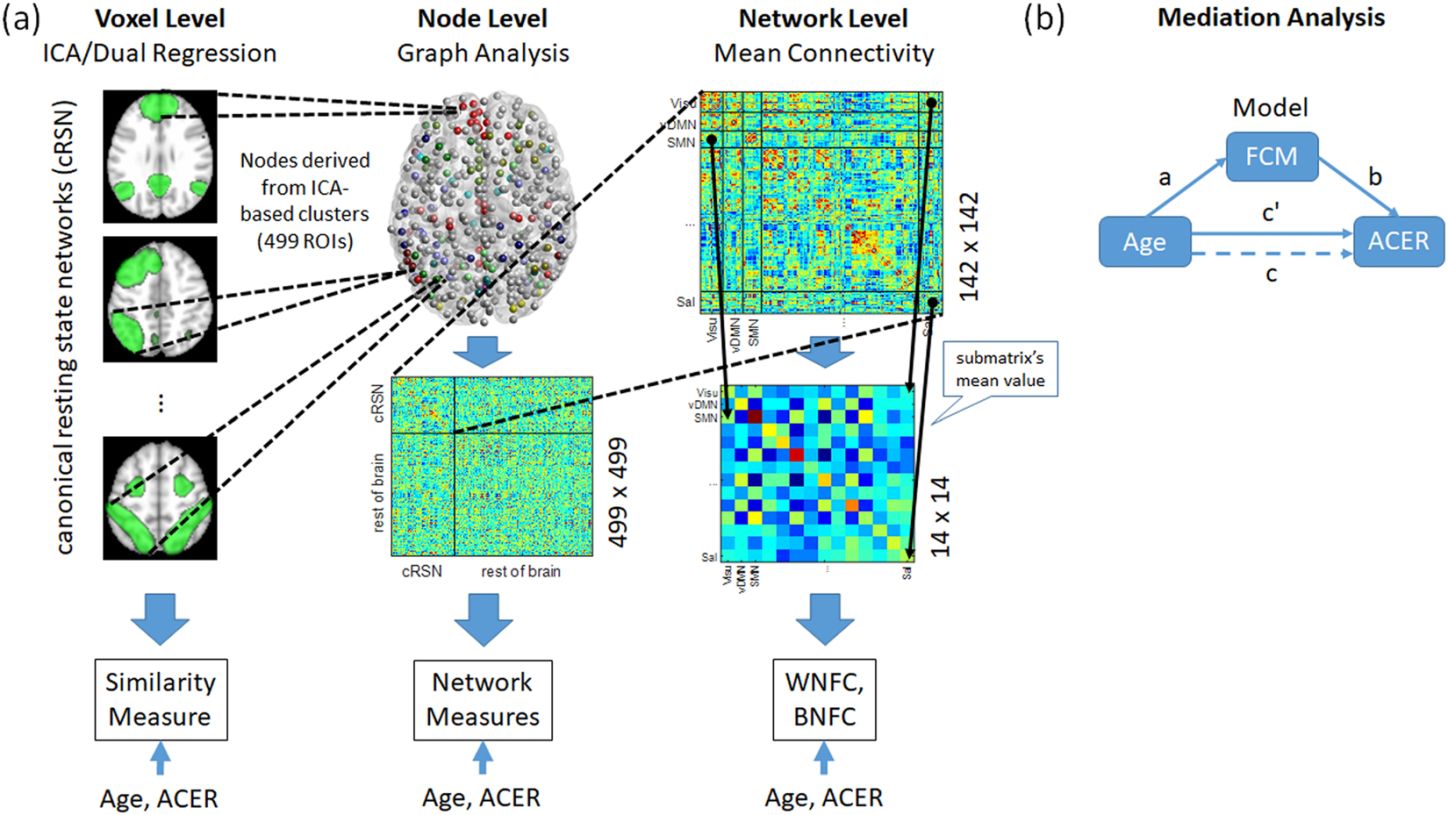
Outline of the overall analysis approach. (**a**) At the voxel level, we used group independent component analysis to extract large-scale canonical resting state networks (cRSNs) from resting state fMRI data and dual regression analysis to identify individual cRSNs. A similarity measure was then estimated for each cRSN relative to a reference RSN. Using clusters derived from cRSNs and the rest of the brain as nodes, we then examined at the node level how the changes observed at the voxel level affected the network properties of the brain using graph analysis. For this, we estimated several whole brain network measures including path length, global efficiency, and degree, among others. Finally, we focused our analysis on the nodes of well-known cRSNs and computed mean connectivity values within cRSNs (within-network functional connectivity, WNFC) and between cRSNs (between-network functional connectivity, BNFC) to investigate changes at the network level. For all estimated measures at all levels, we investigated the measures’ association with age and ACE-R total score. (**b**) For functional connectivity measures (FCM) that showed significant correlation (*p* < 0.05, uncorrected) with both age and ACE-R total score, we further performed mediation analysis to elucidate the relationship among the interacting variables. The mediation model shown in (**b**) was used. Details of the methods used in the analysis are given in the Methods section.

In this study, we aimed to assess age-related reorganization of large-scale functional networks of the brain and identify potential association between this reorganization and individual variability in general cognitive performance. As a measure for general cognitive performance, we used the Addenbrooke’s Cognitive Examination-Revised (ACE-R)^35,36^ total score. ACE-R has been shown as one of the best screening tests for dementia^37^ and its translated versions, including the Japanese version, also showed good to excellent sensitivity and specificity for detecting dementia^38^. We used rsfMRI data from a carefully selected cohort of 129 healthy participants with age ranging from 21 to 86 years old to examine cross sectional changes in the functional organization of several large-scale RSNs over the adult lifespan. As the effects of age could manifest at different spatial or organizational scales, our analysis spanned from the voxel level to the network level to extensively characterize these age-related network changes: (1) We first identify the changes in connectivity patterns in several large-scale RSNs at the voxel level using independent component analysis (ICA) and dual regression^39^. We used ICA to help minimize the effect of head motion and other physiological variables that could affect the estimation of functional connectivity measures as ICA could simultaneously extract both relevant (e.g. RSNs) and structured noise components (e.g., motion-related, physiological, or scanner-induced noise). A global measure estimating the similarity of the connectivity pattern of individual RSNs compared to a given reference was used to further quantify the changes in network integrity and its association with age and general cognitive performance. (2) We then examined at the node level how the changes observed at the voxel level were affecting the whole-brain network topology using graph analysis. We computed several network measures and examined changes in these measures with age and general cognitive performance. In contrast to previous studies, we used a parcellation^40^ that represents functional areas derived directly from nodes of large-scale RSNs rather than using nodes based on structural parcellation (e.g., AAL) or predefined coordinates. (3) We next focused our analysis on how these changes were also affecting the interaction of large-scale RSNs at the network level. For this, we computed the mean connectivity values within and between RSNs to represent network-level connectivity measures and investigated alterations of these measures with age and potential associations with general cognitive performance. (4) Finally, we performed mediation analysis^41^ for functional connectivity measures that showed correlation with both age and general cognitive performance to further elucidate their relationship. Our overall analysis strategy is outlined in Fig. 1 and detailed in the Methods section. By doing this multi-level analysis approach, we aimed to comprehensively investigate age-related network changes and to identify global connectivity measures that may be associated with general cognitive performance.

## Results

### Correlation between age and ACE-R

The correlation between age and ACE-R total score, after controlling for sex, was −0.20 (*p* = 0.027). For the different sub-scores of ACE-R, only attention (*r* = −0.21, *p* = 0.015) and memory (*r* = −0.18, *p* = 0.039) showed correlation with age.

### Age-related voxel-level changes in large-scale canonical RSNs

**Figure 2:**
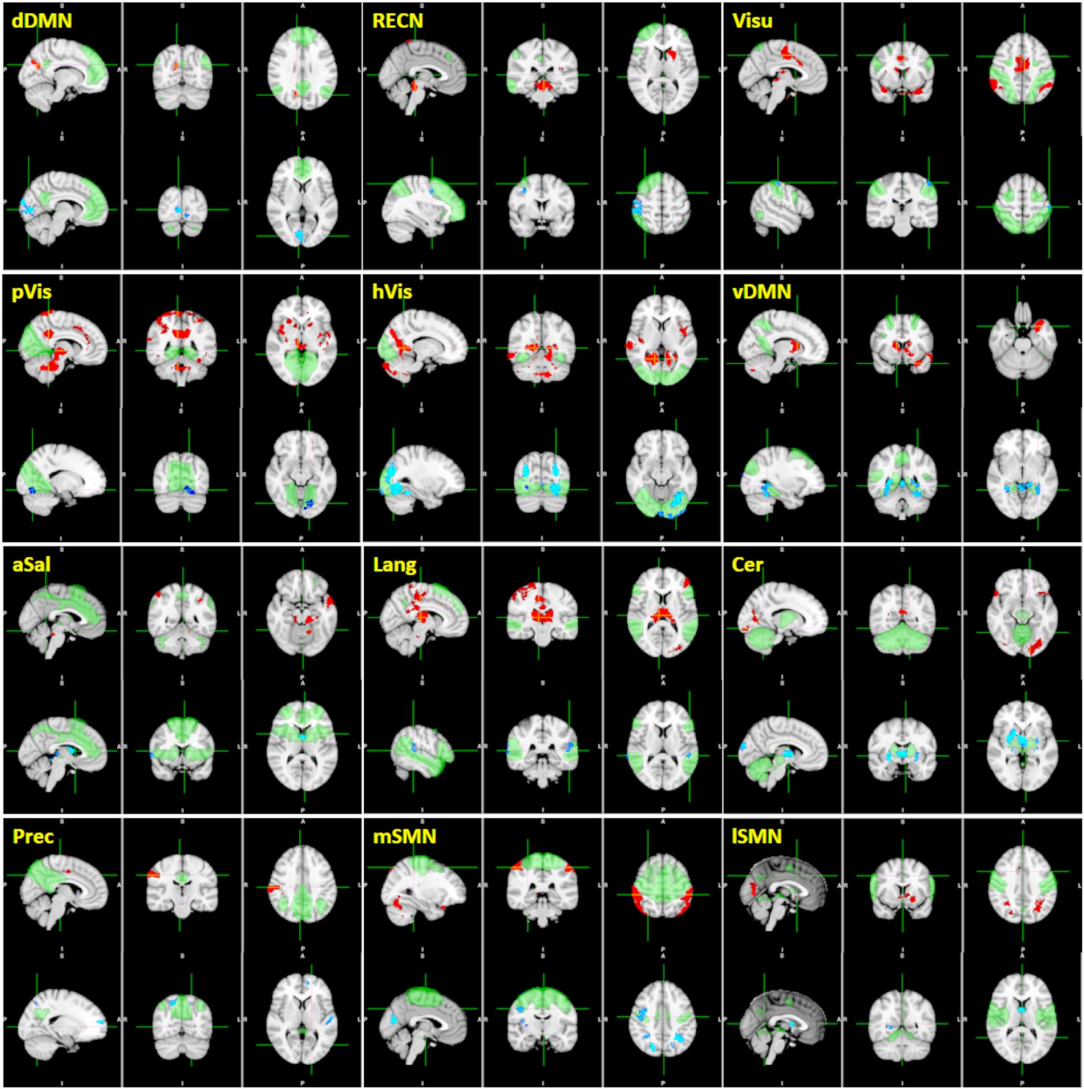
Cross sectional increases and decreases in functional connectivity within and outside canonical resting state networks (RSN) with age. Green regions indicate RSN (group IC thresholded at z > 3.0 for display), blue for negative correlation with age, and red for positive correlation with age. dDMN – dorsal default mode network; RECN – right executive control network; Visu – visuospatial network; pVis – primary visual network; hVis – high visual network; vDMN – ventral default mode network; aSal – anterior salience network; Lang – language network; Cer – cerebellum; Prec – precuneus network; mSMN – medial sensorimotor network; lSMN – lateral sensorimotor network.

To investigate age-related changes in large-scale canonical RSNs at the voxel level, we used group ICA and dual regression analysis. Figure 2 shows voxels with functional connectivity values that significantly correlated with age for some representative canonical RSNs. Green regions indicate the group RSNs obtained using MELODIC. Voxels with connectivity values to the network that showed positive relationship with age are shown in red, while voxels with connectivity values that showed negative relationship are shown in blue. Positive relations were mostly observed in regions outside the network, while negative relations were observed in regions located within and outside the network. Networks that showed widespread positive relation in connectivity with age included primary visual, high visual, medial sensorimotor, visuospatial, language, and ventral default mode, among others, while networks that exhibited negative relation included high visual, medial sensorimotor, cerebellum, and ventral default mode, among others. The list of all clusters showing significant (*p* < 0.05 corrected for multiple comparisons using family-wise error correction) relationship with age for each RSN is given in Supplementary Table S1. These widespread connectivity changes within and outside RSNs suggest an extensive reorganization of the canonical RSNs due to the aging process.

### Similarity measure and its correlation with age and general cognitive performance

To further quantify the observed age-related changes in the connectivity pattern of each RSN, we computed the similarity measure η^2^ of each subject-specific RSN relative to a reference connectivity map to quantify changes in network integrity. The result of the Kolmogorov-Smirnov test for normality of the estimated η^2^ values showed that we could not reject at 5% significance level the null hypothesis that the obtained values come from a normal distribution. Given this, we computed the partial correlations *r* and the corresponding *p*-values between the computed similarity measure and age, sex, and ACE-R total score (Table 1). All RSNs except dorsal default mode, left executive control, and right executive control networks had similarity measures with significant negative partial correlation with age (corrected for multiple comparisons using 5% false discovery rate, FDR q < 0.05). Four of these networks including primary visual, visuospatial, high visual, and lateral sensorimotor also had similarity measures that positively correlated with ACE-R total score (FDR q < 0.05). No RSN had similarity measure that significantly correlated with sex. Results of the mediation analyses (Table 2, Similarity Measure) using the mediation model given in Fig. 1b also showed that the observed correlation in general cognitive performance with age was mediated by the integrity, as quantified by the similarity measure, of several RSNs including primary visual (*ab* = −3.64, *p* = 0.0003), high visual (*ab* = −2.74, *p* = 0.0061), medial sensorimotor (*ab* = −2.39, *p* = 0.017), lateral sensorimotor (*ab* = −2.56, *p* = 0.010), and visuospatial/dorsal attention (*ab* = −2.91, *p* = 0.0037) networks. These alterations in the network integrity of most canonical RSNs quantified using a global measure of similarity further support the observed age-related network reorganization. Moreover, changes in the integrity of some RSNs were also associated with the variability in the general cognitive performance of the participants.

**Table 1:** Partial correlation values *r* of the similarity measure η^2^ with age, sex, and ACE-R total score using reference RSNs constructed from the mean subject-specific RSNs of the young subgroup of participants (age < = 30 years old).

RSN	Age		Sex		ACE-R Total	
	*r*	*p*	*r*	*p*	*r*	*p*
Dorsal DMN	−0.1385	0.1204	0.1394	0.1181	0.1416	0.1122
Primary Visual	**−0.3681**	**0.0000**	−0.0480	0.5922	**0.3466**	**0.0001**
LECN	−0.1399	0.1166	−0.1195	0.1808	0.0688	0.4421
RECN	−0.1332	0.1354	−0.0878	0.3263	0.1725	0.0525
Anterior Salience	**−0.3081**	**0.0004**	0.0659	0.4614	0.0398	0.6567
Visuospatial	**−0.5178**	**0.0000**	0.1378	0.1224	**0.3103**	**0.0004**
High Visual	**−0.3391**	**0.0001**	0.0456	0.6107	**0.2428**	**0.0059**
Precuneus	**−0.2998**	**0.0006**	−0.0699	0.4346	0.0578	0.5189
Language	**−0.3427**	**0.0001**	−0.0365	0.6835	0.1573	0.0775
Medial SMN	**−0.4520**	**0.0000**	0.0520	0.5618	0.2141	0.0156
Cerebellum	**−0.3414**	**0.0001**	0.0440	0.6233	0.1717	0.0536
Lateral SMN	**−0.4146**	**0.0000**	0.0828	0.3545	**0.2440**	**0.0057**
Ventral DMN	**−0.3808**	**0.0000**	0.0788	0.3787	0.1656	0.0628

Highlighted *p*-values are significant after correction for multiple comparisons using 5% false discovery rate (FDR q < 0.05).

RSN – resting state network, DMN – default mode network, LECN – left executive control network, RECN – right executive control network, SMN – sensorimotor network, ACE-R – Addenbrooke’s Cognitive Examination – Revised.

**Table 2:** Results of mediation analysis for the model (Fig. 1b) where a given connectivity measure (similarity, network, or within- and between-network functional connectivity measures) mediated the relationship between age and ACE-R total score.

Model Param	a		b		c’		c		ab	
	z	p	z	p	z	p	z	p	z	p
**Similarity Measure**										
pVis	−3.8796	0.0001	3.6077	0.0003	−0.4874	0.6260	−2.1125	0.0346	−3.6431	0.0003
Visu	−3.4577	0.0005	3.1698	0.0015	0.0178	0.9858	−2.0682	0.0386	−2.9065	0.0037
hVis	−3.8864	0.0001	3.0076	0.0026	−1.1004	0.2711	−2.1487	0.0317	−2.7406	0.0061
mSMN	−3.7305	0.0002	2.7337	0.0063	−0.8887	0.3742	−2.1026	0.0355	−2.3858	0.0170
lSMN	−3.6269	0.0003	2.7589	0.0058	−0.8218	0.4112	−2.0405	0.0413	−2.5633	0.0104
**Network Measures**										
Path length	−3.8565	0.0001	1.5860	0.1127	−0.9646	0.3348	−1.9797	0.0477	−1.9739	0.0484
**WNFC/BNFC**										
Aud	−3.2347	0.0012	3.6457	0.0003	−1.1871	0.2352	−2.1320	0.0330	−3.0275	0.0025
Lang – pVis	−2.0236	0.0430	−2.4032	0.0163	−2.5009	0.0124	−2.1569	0.0310	2.1190	0.0341

Sex was controlled in all regression analyses.

*a* is the coefficient relating the independent variable (age) to the mediator (connectivity measure), *b* is the coefficient relating the mediator to the dependent variable (ACE-R total score), *c*’ is the coefficient relating the independent variable to the dependent variable adjusted for the mediator, *c* is the coefficient relating the independent variable and the dependent variable without mediation, and *ab* is the (mediated) indirect effect. *z* corresponds to the standardized value and *p* the corresponding *p*-value computed using bootstrap method (10,000 samples). pVis – primary visual; Visu – visuospatial; hVis – high visual; mSMN – medial sensorimotor network; lSMN – lateral sensorimotor network; Aud – auditory network; Lang – language network; WNFC – within-network functional connectivity; BNFC – between-network functional connectivity.

### Age-related alterations in whole-brain network topology and its association with ACE-R total score

**Figure 3:**
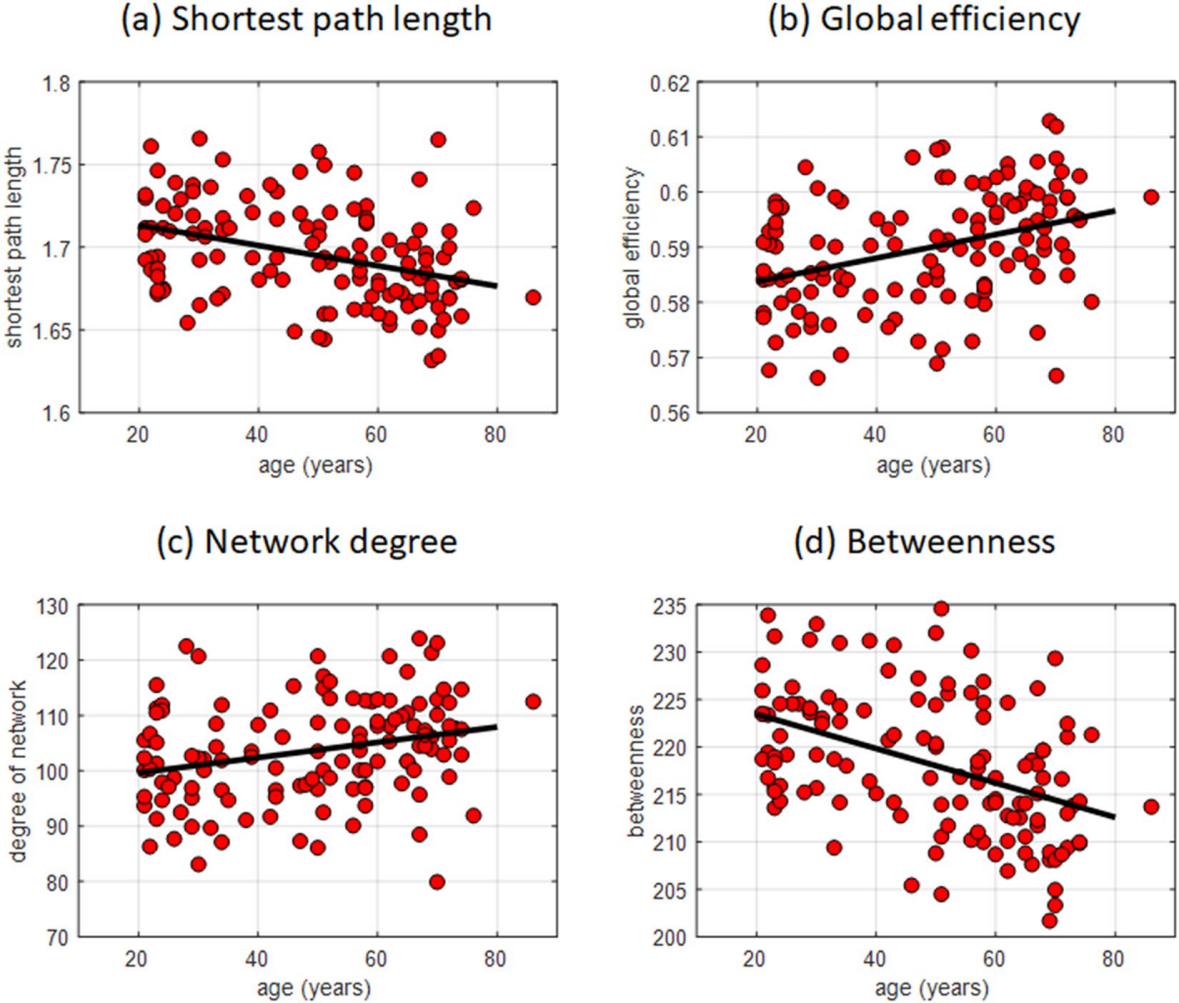
Scatter plots of several network measures exhibiting significant correlation with age after adjusting for sex and ACE-R total score: (**a**) shortest path length (*r* = −0.37, *p* = 2.53 × 10^−5^), (**b**) global efficiency (*r* = 0.37, *p* = 2.24 × 10^−5^), (**c**) degree of network (*r* = 0.29, *p* = 0.0011), and (**d**) betweenness (*r* = −0.42, *p* = 1.03 × 10^−6^). Network measures were estimated using a network-defining correlation threshold set to 0.2.

Next, we examined the effects of the observed age-related connectivity changes in different RSNs to the network topology of the whole brain using graph analysis. Several network measures including path length, global efficiency, network degree, and betweenness, among others, were computed. The Kolmogorov-Smirnov test for normality showed that the null hypothesis that these measures come from a normal distribution could not be rejected at 5% significance level. Figure 3 shows scatter plots of several network properties estimated using graph theory that significantly correlated with age (FDR q < 0.05) for network-defining connectivity threshold value equal to 0.2. We observed a negative correlation between the network’s shortest path length and age (*r* = −0.37, *p* = 2.53 × 10^−5^, Fig. 3a). On the other hand, global efficiency positively correlated with age (*r* = 0.37, *p* = 2.24 × 10^−5^) as shown in Fig. 3b. Similarly, the network degree (Fig. 3c) representing the mean of the degree (number of connections) of all nodes in the network also exhibited positive correlation (*r* = 0.29, *p* = 0.0011), although not as strong as that of the global efficiency. Finally, a more robust decrease in betweenness, a centrality measure representing the fraction of all shortest paths passing through a given node in the network, with age (*r* = −0.42, *p* = 1.03 × 10^−6^) can be observed in Fig. 3d. This association between network measures and age persisted, but appeared to decline, even for higher network-defining thresholds as shown in Table 3 except for network degree, which was no longer correlated with age for threshold values greater than 0.3. In terms of the network measures’ association with ACE-R total scores, the shortest path length, network hierarchy, and betweenness exhibited positive correlation with ACE-R total score, while global efficiency showed negative correlation (FDR q < 0.05) for connectivity threshold value set to 0.2 (Table 4). This association, however, disappeared for higher network-defining connectivity threshold values. Moreover, when adjusting for age and sex, the significance also disappeared. Mediation analyses results only showed mediation effect of path length (*ab* = −1.97, *p* = 0.048) to the relationship between age and ACE-R total score and not as significant as that observed when using similarity measures (Table 2, Network Measures). Together, these findings suggest that the observed reorganization tends to progress towards a more integrated network topology. The association with general cognitive performance, however, was not as robust as compared to that of the other measures.

**Table 3:** Partial correlation values between different network measures and age after adjusting for sex and ACE-R total score. Network measures were estimated using different values of network-defining connectivity threshold *r*.

Network-defining correlation threshold	*r* = 0.2	*r* = 0.25	*r* = 0.3	*r* = 0.35	*r* = 0.40
Shortest path length	−0.37 (2.53 × 10^−5^)	−0.35 (5.24 × 10^−5^)	−0.33 (2.16 × 10^−4^)	−0.29 (8.95 × 10^−4^)	−0.25 (5.23 × 10^−3^)
Global efficiency	0.37 (2.24 × 10^−5^)	0.36 (4.22 × 10^−5^)	0.33 (1.61 × 10^−4^)	0.30 (6.40 × 10^−4^)	0.26 (3.45 × 10^−3^)
Degree	0.29 (0.0011)	0.26 (0.0038)	0.23 (0.012)	NS	NS
Betweenness	−0.42 (1.03 × 10^−6^)	−0.40 (3.71 × 10^−6^)	−0.37 (2.15 × 10^−5^)	−0.34 (1.25 × 10^−4^)	−0.29 (1.00 × 10^−3^)

NS – no significant correlation was observed after correcting for multiple comparisons using FDR q < 0.05. (*) *p*-values.

**Table 4:** Correlation between network properties and ACE-R total score.

Network Measures	Correlation Coefficient (*p*-value)
Shortest path length	0.22 (0.0135)
Global efficiency	−0.22 (0.0127)
Network hierarchy	0.22 (0.0129)
Betweenness	0.27 (0.0021)

Network measures were estimated using a network-defining connectivity threshold value set to *r* = 0.2 (FDR q < 0.05, no adjustment for age and sex).

### Network level age-related changes and its relationship with general cognitive performance

**Figure 4:**
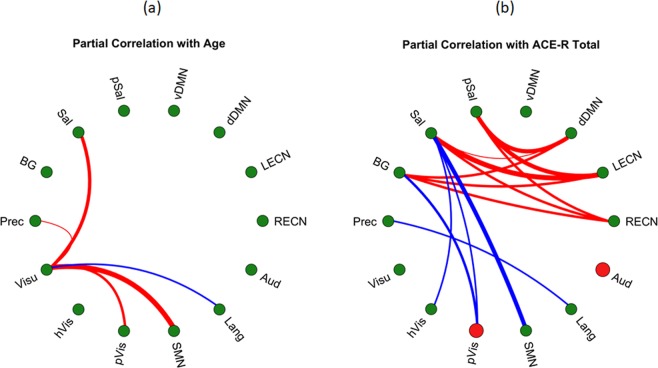
Network graphs showing the correlation between (**a**) network-level functional connectivity and age as well as (**b**) network-level functional connectivity and ACE-R total score. Only significant (FDR q < 0.05) partial correlation values are indicated. Red nodes in (**b**) indicate significant positive partial correlation of within-network functional connectivity (WNFC) with ACE-R total score, while green nodes in both (**a**,**b**) indicate no correlation. Red links between two nodes indicate significant positive partial correlation of between-network functional connectivity (BNFC) with either age (**a**) or ACE-R total score (**b**), while blue links indicate significant negative partial correlation. Line thickness represents the relative strength of the correlation. SMN – sensorimotor network; RECN – right executive control network; Aud – auditory; BG – basal ganglia; dDMN – dorsal default mode network; HVis – high visual; Lang – language; LECN – left executive control network; pSal – posterior salience; Prec – precuneus; PVis – primary visual; Sal – salience; vDMN – ventral default mode network; Visu – visuospatial.

Finally, we also examined network-level measures to identify indications of age-related changes at the network level and its potential association with general cognitive performance. For this, we computed network-level measures corresponding to the mean within-network functional connectivity (WNFC) as well as the mean between-network functional connectivity (BNFC). Figure 4a shows the relationship between WNFC/BNFC and age. At the network level, only the visuospatial network’s BNFC values showed significant (FDR q < 0.05) association with age. Negative partial correlation (blue line) was observed in the BNFC of visuospatial and language networks with age. On the other hand, positive partial correlations between BNFC and age (red lines) were observed in visuospatial with sensorimotor, primary visual, precuneus, and salience networks. These findings further support the results using ICA and dual regression analyses signifying the widespread reorganization of canonical RSNs with age with increasing between-network connectivity.

In terms of general cognitive performance, Fig. 4b shows the relationship between WNFC and BNFC with ACE-R total score after accounting for the contribution of age and sex using partial correlations. Independent of age and sex, significant (FDR q < 0.05) positive partial correlation was observed between WNFC of the primary visual and auditory networks with ACE-R total score as indicated by the red nodes in Fig. 4b. The mean connectivity values among (left/right) executive control, (posterior) salience, and dorsal default mode as well as (left/right) executive control, basal ganglia, and dorsal default mode were also positively correlated with ACE-R total score. On the other hand, the mean connectivity values between (primary/high) visual and sensorimotor with salience, precuneus with language, as well as primary visual with basal ganglia were negatively correlated with ACE-R total score. Generally, the BNFC among core neurocognitive networks (salience, dorsal default mode, and executive control) and basal ganglia positively correlated with ACE-R total score, while that between core neurocognitive networks/basal ganglia and primary processing networks (visual and sensorimotor) negatively correlated with ACE-R total score. Results of the mediation analyses showed that the WNFC of the auditory network and the BNFC between language and primary visual networks mediated the relationship between age and ACE-R total score (*ab* = −3.03, *p* = 0.0025 and *ab* = 2.12, *p* = 0.034, respectively; see Table 2, WNFC/BNFC).

## Discussion

We examined cross sectional changes in the organization of large-scale functional networks in the brain over the adult lifespan using rsfMRI data from a carefully selected cohort of healthy participants in order to reduce factors that may influence the reliability of the results. In addition, we investigated the association between the estimated functional connectivity measures and ACE-R total score to identify potential connectivity-related correlates of the individual differences in general cognitive performance observed across participants.

Converging results from multiple analyses showed large-scale reorganization of functional brain networks with advancing age. In particular, (1) positive correlation between functional connectivity and age was mostly observed between networks, while negative correlation was observed both within and between networks; (2) compared with reference connectivity maps derived from the mean RSNs of the young adult subgroup, all canonical RSNs except the left and right executive control and dorsal default mode networks had similarity measures that negatively correlated with age; and (3) network measures estimated using graph theory showed progression towards a more integrated network topology as indicated by a decreasing shortest path length and an increasing global efficiency with age. In terms of the connectivity’s association with general cognitive performance, (4) primary processing networks including (primary/high) visual and (lateral) sensorimotor networks, and the visuospatial network have similarity measures that positively correlated with ACE-R total score suggesting the potential relevance of the integrity of these networks in maintaining normal cognitive function. Moreover, (5) between-network connectivity among higher level cognitive networks including executive control, salience, and (dorsal) default mode networks, and basal ganglia network were significantly correlated with ACE-R total score, implicating the potential role of the interaction between these networks in the observed individual differences in general cognitive performance. Interestingly, the interconnections between these networks were minimally affected by age-related connectivity alterations. Since aberrant functioning and organization of these networks have been implicated in several psychiatric and neurological disorders^42^, the relative preservation of the connectivity within and between these networks may be indicative of the importance of these connections in preserving cognitive function during healthy aging. Finally, (6) results of the mediation analyses further indicated that the observed relationship between aging and the relative decline in general cognitive performance could be mediated by changes in relevant functional connectivity measures.

Previous studies have shown alterations in functional connectivity of canonical RSNs, reflecting functional network reorganization (e.g., see Sala-Llonch and colleagues^43^ for a review). Our results show that generally, within-network connectivity values tended to be lower in advanced age, while between-network connectivity values tended to be higher. This general observation is consistent with the findings of other studies that have been reported in the literature^28,29,44^. In particular, the reported decrease in within-network connectivity in the executive control and visual networks and increase in between-network connectivity in the dorsal attention/visuospatial, sensorimotor, and salience networks^28^ are consistent with our findings (see Figs 2 and 4). Other studies also reported decreased within-network connectivity in executive control^31^, visual^45^, and default mode^24,27,31^ networks consistent with our voxel-level results. Discrepancies in the details of the reported changes in other studies may be present but the general observation remains the same. These differences could be attributed to variations in defining the network (e.g., using ICA or pre-defined nodes), in defining nodes (using fixed MNI coordinates, or data-driven such as clusters derived from ICA decomposition, or other parcellations), and in methods defining connectivity/network measures (e.g., using seed-based, ICA, or graph theory), among others.

Results of the network analysis using whole brain parcellation also reflect greater functional reorganization in the brain that tended to progress towards a more integrated network. These findings are consistent with the observed increased in connectivity with age with regions outside the network at the voxel level. Measures that can be used to quantify network integration include shortest path length, global efficiency, and node degree, among others. Decrease in shortest path length with age indicates that information travels from one node to another using shorter path, suggesting that the network becomes more integrated. This is further supported by the observed increase in global efficiency (GE), indicating a more efficient pathway from one node to another. Previous studies had also examined differences in GE in young and older adults and found similar results^33,46^. Higher level functions that require integration of information from different sources could benefit from the increase in global efficiency across the whole network^47^. The observed increase in network degree also means more nodes were linked together and decrease in betweenness also suggests less clustering of nodes, again pointing to a higher network integration with age. Intriguingly, the overall increase in global efficiency or whole-brain network integrity was associated with a negative correlation in ACE-R total score (Table 4), which appeared to be contradictory. However, most of the connectivity increases we observed were driven by the primary processing systems. It is therefore not surprising if these increases in between-network connectivity drove the overall increase in global efficiency (or whole-brain network integration). These connectivity increases, however, were also associated with the decrease in these networks’ integrity, which in turn, was associated with general cognitive performance. Thus, in spite of the increased in global efficiency with age, the overall effect in general cognitive performance was negative.

At the network level, only the visuospatial network, also known as the dorsal attention network, exhibited a more widespread cross sectional increased connectivity towards other networks (Fig. 4a). This is also reflected in the estimated similarity measure (voxel-level analysis) relative to that of the young adult subgroup, which showed negative correlation with age. This suggests a weakening network integrity, which could be driven by the increase in connectivity with nodes from other networks. Association systems typically direct and integrate information from a wide range of tasks and across modalities. It is therefore not surprising that the connectivity of the visuospatial network with sensorimotor and visual processing networks (sensorimotor, visual, and basal ganglia networks) as well as other association systems (salience and precuneus) would increase with age. What is intriguing is that the estimated similarity measure of this network positively correlated with ACE-R total score suggesting that the preservation of the integrity of this network with age may be important for the individual’s overall cognitive performance. This observation is further supported by the result of the mediation analysis where the similarity measure of this network had significant mediation effect on the relationship between aging and general cognitive performance.

Like the visuospatial network, the precuneus, which is a subsystem of the classical default mode network, also exhibited tendency towards higher connectivity with other networks (visuospatial, right executive control, and salience networks) with advanced age accounting for the increasing dissimilarity of the connectivity pattern relative to that of the young adult subgroup. However, unlike the visuospatial network, we did not observe significant correlation between the similarity measure and ACE-R total score. Moreover, its connectivity with other networks was also not correlated with ACE-R total. This result seemed to contradict several studies showing the involvement of the precuneus/posterior cingulate cortex in Alzheimer’s disease (AD) and connectivity alterations in this region in AD are often associated with cognitive decline^48–50^. In light of this, we speculated that the observed increased connectivity with age in the precuneus could be a form of compensatory mechanism in support to other networks given that this region is also a major connectivity hub in the brain. In this cohort, all participants are cognitively normal and therefore should have a well-functioning compensatory mechanism especially in advanced age. We could therefore interpret findings related to AD with regards to the precuneus as a possible failure of compensation, which could then lead to cognitive decline. A recent paper by Schultz and colleagues^51^ showing an initial hyperconnectivity phase in the default mode and salience networks in the course of preclinical AD, then followed by a hypoconnectivity phase, could lend support to this interpretation. Moreover, Jones and colleagues^52^ also proposed that failure in the posterior default mode (equivalent to the precuneus network) could trigger a series of downstream network failures leading to Alzheimer’s disease. Elucidating the role of the precuneus network in cognition during healthy aging is currently beyond the scope of this paper.

In terms of general cognitive performance, we observed that the relatively well-preserved between-network connections of the higher level cognitive networks were positively correlated with ACE-R total score (Fig. 4b). This could imply that better general cognitive performance is closely associated with the strong interaction among default mode, salience, and executive control networks (red solid lines). These 3 RSNs have been considered as core neurocognitive networks due to the critical role these networks play across a wide range of cognitive tasks^23,42^. The default mode network, usually de-activated during task performance, has been linked with internally oriented or self-related processes such as stimulus-independent thoughts^53^, mind wandering^54^, introspection^55^, and social cognition^56^, among others, while the executive control network, activated during cognitively demanding tasks, is associated with the active maintenance and manipulation of information in working memory and in making judgment and/or decision during goal-directed behavior^57,58^. On the other hand, the salience network has been implicated to play a role in switching between the activation and deactivation of the externally oriented executive control network and the internally oriented default mode network^59,60^. Our findings identifying the connectivity between these networks being correlated with general cognitive performance reinforces the significance of the role these networks play in general cognition.

Aside from the core neurocognitive networks, our results also suggested the potential role of the interaction between basal ganglia and executive control networks as well as between basal ganglia and default mode networks for the maintenance of general cognition. This relationship is akin to that of the core neurocognitive networks with the basal ganglia network switching place with the salience network. This interaction is not without precedent and previous studies have shown examples of the interaction among these 3 networks. Using intra/extradimensional set-shifting tasks measuring cognitive flexibility, Vatansever and colleagues^61^ have shown that participants with greater functional connectivity between default mode and basal ganglia achieved better task performance. Anatomically, the striatum is also known to have reciprocal connections with the dorsolateral prefrontal cortex (dlPFC) and the posterior parietal cortex (PPC)^62,63^, regions belonging to the executive control network. In Parkinson’s disease (PD), which primarily affects the striatum, the dlPFC and PPC have been shown to display abnormal activity during cognitively demanding tasks^64,65^ and decrease in connectivity between the striatum and the default mode network, shown to correlate with the symptom’s severity, has also been reported^66^. Given that the interaction among these 3 networks also correlated with ACE-R total score in this study could lend support to the possible role of basal ganglia network in general cognition.

Compared to the core neurocognitive networks, the primary processing networks’ integrity, rather than between-network connectivity, is associated with general cognitive performance. The estimated similarity measures of these networks negatively correlated with age, suggesting the connectivity patterns are becoming more dissimilar compared to the reference. These changes could be driven by the networks’ increased connectivity with regions outside the network (in particular, with nodes of the visuospatial network as shown in Fig. 4a). Moreover, this changing connectivity pattern is also positively correlated with ACE-R total score suggesting the potential role of the integrity of these networks in maintaining normal cognitive performance. As an example, recent work from our group^67^ showed that non-demented PD patients with multiple cognitive domain deficits exhibited greater reductions in connectivity in the visual network compared to healthy controls. Mediation analysis further confirmed the role of the integrity of these networks on the relationship between aging and ACE-R total score. In addition to the similarity measure, the connectivity of these networks with the salience and basal ganglia networks also negatively correlated with ACE-R total score, an indication that direct integration of these networks to the core neurocognitive networks during aging could be maladaptive.

Finally, we would like also to address the issue on the use of ACE-R total score as the main measure for general cognitive performance. Here, our aim was not to examine sources of global cognition per se, which is beyond the scope of the paper, but rather to investigate whether the observed changes in measures of functional connectivity (similarity index, network measures, BNFC, and WNFC) could be associated with general cognitive performance. There are several tests currently in use to measure general cognition. Among these cognitive tests, we used ACE-R total score. ACE-R is a brief battery providing evaluation of six cognitive domains, could take about 20 minutes to complete, includes the Mini Mental State Examination (MMSE)^68^ with the additional non-MMSE items shown to improve estimates of cognitive ability by 16%^69^ and with lower ceiling effect (as compared to MMSE). ACE-R has also been shown to have the best diagnostic performance (sensitivity and specificity) for dementia^37^ including its various translations. Since aging is a main risk factor for several neurodegenerative disorders leading to dementia, we consider the use ACE-R total score as a measure of general cognitive performance reasonable. Recent studies^70,71^ have also started looking at age-related neurological disorders as “syndromes of accelerated brain aging process.” Viewed this way, using a cognitive measure that would span the “brain aging” spectrum (from healthy aging to mild cognitive impairment to dementia) may also be helpful in identifying relevant connectivity-based biomarkers that could be associated with cognitive dysfunction.

## Limitations

In this study, all participants are cognitively normal with ACE-R total score above 88. The observed connectivity-related correlates of the variability in general cognitive performance may therefore be limited to normal cognition. Studies involving clinical population would be needed to verify whether the observed association between functional connectivity and cognitive performance has general applicability and are predictive of any age-related cognitive decline. However, some studies cited above do indicate that this association may have more general applicability even in neurodegenerative cases and may serve as an important template in identifying the relevant connectivity correlates that could lead to cognitive degeneration. Another limitation of the study is the sample size. The current sample size may not be adequate considering the range of age being investigated. However, we did strive to have each decade of age reasonably represented except for the 8^th^ decade, which has only 1 participant. In addition, there is also an imbalance in the number of male and female participants with more of the latter than the former. This could be a reflection of the overall proportion of men and women in the general population given the longer average life expectancy of women (87.26 years) as compared to men (81.09 years) in Japan. The higher stroke prevalence, whether ischemic or hemorrhagic, in men also led to the higher number of excluded male participants compared to females. Other contributing factors include availability as well as interest in participating research studies. To account for this difference, we included sex as a co-variate/regressor in most analyses to minimize its potential effects in our results. The presented results should therefore be interpreted in view of these limitations.

## Conclusion

We examined the relationship between functional connectivity in large-scale brain networks and age over the adult lifespan. Our results showed significant alterations in the functional connectivity patterns characterized by weaker within-network connectivity and higher between-network connectivity in advanced age. Estimated network properties also tend to suggest a progression towards a more integrated network topology as the brain aged. Between-network interactions among the so-called core neurocognitive networks plus the basal ganglia network, integrity of within-network connectivity pattern, as estimated by the similarity measure η^2^, of primary processing networks and visuospatial network and possible compensatory action of the precuneus network were all associated with general cognitive performance. Taken together, these findings provide further evidence supporting age-related reorganization of large-scale functional brain networks and its potential association with general cognitive performance during healthy aging.

## Methods

### Participants

For this study, healthy volunteers from Nagoya City and neighboring areas were recruited through public relations efforts and information dissemination campaigns via posters, flyers, and word-of-mouth advertising in collaboration with local communities. As a consequence, our healthy volunteers participated in this aging cohort study^4^ at their own initiative. A total of 445 healthy adults volunteered from July 2014 to March 2016. Japanese board-certified neurologists, neurosurgeons, and speech therapists performed clinical assessments that included the Mini-Mental State Examination (MMSE)^68^ and the Japanese version of Addenbrooke’s Cognitive Examination-Revised (ACE-R) for evaluating general cognitive performance^35,36^ and the Beck Depression Inventory for mood disturbances^72^. Although we also assessed MMSE, it was only used for additional screening of the participants. Demographic data including education, past medical history, medication, drinking and smoking habits, and family history of neurodegenerative diseases were also collected. The study conformed to the Ethical Guidelines for Medical and Health Research Involving Human Subjects as endorsed by the Japanese Government and was approved by the Ethics Committee of Nagoya University Graduate School of Medicine. All participants gave written informed consent before joining the study.

Of the 445 participants initially recruited, 6 were unable to complete the ACE-R and were not scanned, 84 were excluded due to asymptomatic cerebral infarction or hemorrhage, benign brain tumor, leptomeningeal cyst, cavum septum pellucidum, cavum vergae, and apparent motion artifact, and an additional 44 were further excluded due to obvious brain atrophy or WM abnormalities characterized by hyperintensities in T2-weighted images that were grade 2 or 3 based on the Fazekas hyperintensity rating system. Of the remaining 311 participants, 16 had MMSE scores less than 26 or ACE-R score less than 89 and 2 had incomplete imaging data and were also excluded. To control for the effect of head motion, a critical confounder in the computation of functional connectivity measures using rsfMRI data, we further performed additional screening based on the amount of head motion measured using framewise displacement (FD)^73^. We rigorously excluded the datasets with mean FD equal or greater than 0.2 mm and the number of volumes with FD > 0.2 mm was more than 20% of the total number of volumes. With this very strict head motion criterion, 164 participants were further excluded. The final number of participants included in the analysis was 129 with age ranging from 21 to 86 years old. The participants’ characteristics are summarized in Table 5.

**Table 5:** Participants’ characteristics.

Age Range	Count	M	F	Mean MMSE (SD)	Mean ACE-R (SD)					
					Total	Attn	Mem	Flncy	Lang	Visu
20–29	28	13	15	29.9 (0.4)	96.7 (1.9)	18.0 (0.0)	24.3 (1.1)	13.9 (0.4)	24.8 (1.3)	15.8 (0.6)
30–39	16	4	12	30.0 (0.0)	97.9 (2.4)	18.0 (0.0)	24.9 (1.3)	13.6 (1.1)	25.4 (0.9)	16.0 (0.0)
40–49	12	4	8	29.6 (0.5)	96.9 (2.8)	18.0 (0.0)	24.2 (2.2)	13.6 (1.0)	25.2 (0.9)	15.8 (0.6)
50–59	26	4	22	29.7 (0.5)	97.5 (1.7)	18.0 (0.2)	24.7 (1.5)	13.7 (0.6)	25.3 (0.7)	15.8 (0.5)
60–69	30	8	22	29.4 (0.7)	96.5 (2.5)	17.9 (0.4)	23.8 (2.3)	13.8 (0.6)	25.2 (0.8)	15.8 (0.8)
70–79	16	3	13	28.9 (1.0)	95.1 (3.0)	17.9 (0.3)	23.5 (2.0)	13.4 (1.0)	24.8 (1.1)	15.6 (0.7)
80–89	1	0	1	28.0 (-)	91.0 (-)	17.0 (-)	20.0 (-)	14.0 (-)	25.0 (-)	15.0 (-)
Total	129	36	93							

M – male; F – female; MMSE – Mini-Mental State Examination; ACE-R – Addenbrooke’s Cognitive Examination-Revised; Attn – attention; Mem – memory; Flncy – fluency; Lang – language; Visu – visuospatial; SD – standard deviation.

### MRI data

All MRI scans were performed using a Siemens Magnetom Verio (Siemens, Erlangen, Germany) 3.0 T scanner with a 32-channel head coil at Nagoya University’s Brain and Mind Research Center. For each participant, a high resolution T1-weighted (T1w) image was acquired using a 3D MPRAGE (Magnetization Prepared Rapid Acquisition Gradient Echo, Siemens) pulse sequence^74^. The imaging parameters used were as follows: repetition time (TR)/MPRAGE TR = 7.4/2500 ms, echo time (TE) = 2.48 ms, inversion time (TI) = 900 ms, 192 sagittal slices with a distance factor of 50% and 1-mm thickness, FOV = 256 mm, 256 × 256 matrix dimension, in-plane voxel resolution of 1.0 × 1.0 mm^2^, flip angle (FA) = 8 degrees, and total scan time equal to 5 min and 49 s. Aside from T1w images, rsfMRI data were also acquired using an ascending gradient echo (GE) echo planar imaging (EPI) sequence with the following imaging parameters: TR = 2.5 s, TE = 30 ms, 39 transverse slices with a 0.5-mm inter-slice interval and 3-mm thickness, FOV = 192 mm, 64 × 64 matrix dimension, FA = 80 degrees, and total scan time of 8 min and 15 s. Participants were instructed to close their eyes but not to fall asleep during the scan. Other MRI scans were also acquired during the same imaging session, but the analysis of these images will be reported elsewhere.

### Image preprocessing

The anatomical T1w and rsfMRI data were preprocessed using Statistical Parametric Mapping (SPM12, Wellcome Trust Center for Neuroimaging, London, UK) running on Matlab (R2016a, MathWorks, Natick, Mass, USA). The T1w images were first segmented into component images including gray matter (GM), white matter (WM), and cerebrospinal fluid (CSF), among others, using SPM12’s segmentation approach^75^. Bias-corrected T1w images together with the transformation information from subject space to MNI (Montreal Imaging Institute) space were then obtained. For each rsfMRI dataset, we excluded the first 5 volumes in the series to account for the initial scanner inhomogeneity. The remaining volumes were then slice-time corrected relative to the middle slice (slice 20), and then realigned relative to the mean functional volume. The mean volume, together with the realigned functional images, were then co-registered to the bias-corrected T1w image. Using the transformation obtained during segmentation, the functional images were normalized to the MNI space, resampled to have an isotropic voxel resolution equal to 2 × 2 × 2 mm^3^, and smoothed using an isotropic 8-mm full-width-at-half-maximum (FWHM) 3D Gaussian filter. To correct for head motion and contribution from other nuisance signals, we regressed out 24 motion-related regressors [R_t_ R_t_^2^ R_t−1_ R_t−1_^2^], where R = [x, y, z, roll, pitch, yaw] represents the estimated motion parameters (3 translations and 3 rotations). Signals extracted from spherical ROIs within the CSF (center’s MNI coordinate = [20, −32, 18], radius = 4 mm) and WM (center’s MNI coordinate = [24, −12, 34], radius = 4 mm), the global signal, as well as the signals’ derivatives were also removed. Finally, the preprocessed data were then bandpass filtered within 0.01–0.1 Hz. These additional preprocessing were performed using in-house Matlab scripts. The resulting dataset were used in the succeeding analysis.

### Analysis of large-scale canonical RSNs

To extract the canonical RSNs from the preprocessed rsfMRI dataset, we used group independent component analysis (ICA) as implemented in MELODIC, a component software from the FSL package^76^. All preprocessed rsfMRI datasets were temporally concatenated and 20 spatial independent components (ICs) were extracted. We only extracted 20 ICs to minimize the fractionation of the canonical RSNs into several ICs. ICs with significant overlapped with established RSN templates^77^ including dorsal and ventral default mode network, left and right executive control networks, salience network, primary and high visual networks, lateral and medial sensorimotor network, language network, visuospatial network (also known as dorsal attention network), precuneus network, and cerebellum network were then identified. A dual regression analysis^39^ was then performed to extract subject-specific RSNs using the group ICs. Briefly, the extracted group ICs were used as spatial regressors for each participant’s rsfMRI dataset and regression parameters were estimated at each time point. This gave a series of parameters associated with each group IC. The computed parameter time series for all ICs were then entered into a second regression analysis to generate subject-specific RSNs associated with the group RSNs.

We then performed regression analyses with age and sex as regressors using the computed subject-specific RSNs. We used FSL’s randomise tool^78,79^ to fit the model and compute the model parameters’ significance using a nonparametric permutation testing with 5000 permutations. A threshold-free cluster enhancement (TFCE) technique^80^ was applied in the resulting statistical images. The final statistical maps were corrected for multiple comparisons using family-wise error (FWE) correction with *p* < 0.05.

### Estimation and analysis of the similarity measure

To measure the integrity of each RSN, a global measure quantifying changes in the connectivity pattern of each RSN relative to a reference was also computed. For this, we used a spatial similarity measure given by η^2^ ^81,82^:

$${\eta }^{2}=1-\frac{{\sum }_{i=1}^{V}\,[{({a}_{i}-{m}_{i})}^{2}+{({b}_{i}-{m}_{i})}^{2}]}{{\sum }_{i=1}^{V}\,[{({a}_{i}-\bar{M})}^{2}+{({b}_{i}-\bar{M})}^{2}]}$$

where *a*_i_ and *b*_i_ are values at voxel *i* in connectivity maps *a* and *b*, respectively, *m*_i_ is the mean value of the two maps at voxel *i*, $\bar{M}$ is the grand mean value across the mean image *m*, and *V* is the total number of voxels in the maps. Here, *a* represents a subject-specific RSN (e.g., ventral DMN) and *b* is the corresponding reference RSN (e.g., reference ventral DMN). For reference RSNs (RSN_REF_), we used the mean of subject-specific RSNs from a subgroup of young adults (age < = 30 years old, *N* = 31 participants). The choice of 30 years old as the cut-off age to generate the reference RSNs was arbitrary but was motivated by the assumption that at this stage of development, the RSNs are optimally developed and could therefore serve as a good reference for changes occurring during the aging process. We also performed the same analysis using 40 years old as the cut-off age and obtained approximately the same findings and so will not be presented in the results. Finally, we note that η^2^ can vary from 0 to 1, with 0 indicating no similarity and 1 being identical. This measure enables the quantification of the similarity, not just the correlation, between two images. Moreover, this measure can also capture within- and between-network connectivity pattern differences and is therefore suitable for assessing alterations in the connectivity pattern of large-scale RSNs. The relations between the estimated η^2^ and age, sex, and ACE-R total score were computed using partial correlations as implemented in Matlab’s *partialcorri*() function. Correlations were considered significant after correcting for multiple comparisons using a 5% false discovery rate (FDR q < 0.05). We further verified whether the estimated values of η^2^ were normally distributed using the Kolmogorov-Smirnov test as implemented in Matlab’s *kstest*() function.

### Graph analysis of whole-brain network topology

Aside from canonical RSN analysis, we also performed whole brain connectivity analysis to investigate age-related connectivity changes across the entire brain at the cluster/node level and identify alterations in whole-brain network topology. For this, we used a parcellation that divided the brain into 499 ROIs^40^. This parcellation extended the original 90 functional ROIs (https://findlab.stanford.edu/functional_ROIs.html) representing the nodes of 14 RSN templates^77^ obtained using group ICA by further subdividing very large nodes in the RSN templates and adding more ROIs to cover the rest of the cortex and subcortical areas. Of the 499 ROIs, 4 were from the auditory network, 7 from basal ganglia, 21 from dorsal default mode, 13 from ventral default mode, 4 from high visual, 9 from language, 10 from left executive control, 14 from right executive control, 12 from sensorimotor, 13 from posterior salience, 6 from precuneus, 2 from primary visual, 12 from anterior salience, and 15 from visuospatial/dorsal attention network. The remaining 357 ROIs were from the rest of the cortex and other subcortical areas. A complete list of the 499 ROIs including MNI coordinates, cluster sizes, and labels is given in Supplementary Table S2.

BOLD signals from all voxels within a given ROI were extracted and averaged. Each ROI represented a node in the whole-brain network and the averaged BOLD signal as the node’s time series. The connectivity matrix for each participant was then computed using Pearson’s correlation coefficient between the time series of pairs of nodes giving a 499 × 499 connectivity matrix. From the computed connectivity matrix, several network measures, including shortest path length, global efficiency, network degree, betweenness, and modularity, among others, were estimated using GRETNA^83^. Note that the resulting values of these measures could depend on the value used to threshold the connectivity matrix to define the network. Consequently, we used several network-defining connectivity threshold values given by *r* = 0.20, 0.25, 0.30, 0.35, and 0.40 to validate the consistency of our findings across different threshold levels. The correlation between the computed network measures and age, sex, and ACE-R total score was estimated using Matlab’s *partialcorri*() function. Correlations were considered significant after correcting for multiple comparisons using FDR q < 0.05. Testing for normality of the different network measures was also performed using Kolmogorov-Smirnov test as implemented in Matlab’s *kstest*() function.

### Estimation and analysis of network-level connectivity measures

To examine the relationship between functional connectivity and age at the network level, we further assessed the connectivity within and between canonical RSNs using a subset of nodes (142 nodes from the 14 RSNs) from the 499 ROIs. For this analysis, we transformed the estimated correlation coefficients into z-scores, representing connectivity values, using Fisher’s transform. We then computed for each RSN the within-network functional connectivity (WNFC), which represents the mean of all connectivity values between nodes within the same RSN. Furthermore, we also computed the between-network functional connectivity (BNFC) represented by the mean of all connectivity values between nodes in one network and that in another network. We then computed the correlation between WNFC and age as well as between BNFC and age after controlling for sex and ACE-R total score using partial correlations. We also assessed the relationship between WNFC and ACE-R total score as well as BNFC and ACE-R total score after controlling for age and sex. This is implemented using Matlab’s *partialcorri()* function. Correlation values were considered significant after correcting for multiple comparisons using FDR q < 0.05.

### Mediation analysis

To further elucidate the relationship among the different functional connectivity measures, age, and general cognitive performance, we performed mediation analysis^41^ for functional connectivity measures that showed correlation (*p* < 0.05, uncorrected) with age and with ACE-R total score. We tested the model where age was used as the independent variable, the functional connectivity measure (similarity measure, network properties, WNFC, or BNFC) as the mediator, and ACE-R total score as the dependent variable (Fig. 1b). We note that alternative models (e.g., the connectivity measure was used as the independent variable, age as the mediator, and ACE-R total score as the dependent variable) were also considered for completeness but did not show significant mediation effect and therefore would not be discussed further. We used the Mediation Toolbox^84^ (https://github.com/canlab/MediationToolbox) to estimate the different model parameters. Sex was controlled in all regression analyses and *p*-values were estimated using bootstrap method (10,000 samples).

## Supplementary information

Supplementary Information

## Data Availability

The datasets generated and analyzed in the current study are not publicly available due to privacy and legal reasons. The data are however available from the corresponding author upon reasonable request and with permission from the Ethics Committee of Nagoya University Graduate School of Medicine.
