# Supplementary material for: Reorganization of brain networks and its association with general cognitive performance over the adult lifespan
Source: Sci Rep. 2019 Aug 6;9:11352. doi: 10.1038/s41598-019-47922-x (PMC6684569; doi:10.1038/s41598-019-47922-x)
Supplement: Supplementary file 1 — Supplementary Information [file 41598_2019_47922_MOESM1_ESM.pdf]

Reorganization of brain networks and its association with general cognitive performance over the adult lifespan

Epifanio Bagarinao[1], Hirohisa Watanabe[1,2,8,*], Satoshi Maesawa[1,3], Daisuke Mori[1], Kazuhiro Hara[2], Kazuya Kawabata[2], Noritaka Yoneyama[2], Reiko Ohdake[2], Kazunori Imai[2], Michihito Masuda[2], Takamasa Yokoi[2], Aya Ogura[2], Toshiaki Taoka[4], Shuji Koyama[1], Hiroki C. Tanabe[5], Masahisa Katsuno[2], Toshihiko Wakabayashi[3], Masafumi Kuzuya[6], Norio Ozaki[7], Minoru Hoshiyama[1], Haruo Isoda[1], Shinji Naganawa[1,4], Gen Sobue[1,*]

[1]Brain and Mind Research Center, Nagoya University, Nagoya, Aichi, Japan

[2]Department of Neurology, Nagoya University Graduate School of Medicine, Nagoya, Aichi, Japan

[3]Department of Neurosurgery, Nagoya University Graduate School of Medicine, Nagoya, Aichi, Japan

[4]Department of Radiology, Nagoya University Graduate School of Medicine, Nagoya, Aichi, Japan

[5]Department of Cognitive and Psychological Sciences, Graduate School of Informatics, Nagoya University, Nagoya, Aichi, Japan

[6]Department of Community Healthcare and Geriatrics, Nagoya University Graduate School of Medicine and Institutes of Innovation for Future Society, Nagoya University, Nagoya, Aichi, Japan

[7]Department of Psychiatry, Nagoya University Graduate School of Medicine, Nagoya, Aichi, Japan

[8]Department of Neurology, Fujita Health University School of Medicine, Toyoake, Aichi, Japan

[*)] Address correspondence to:

Gen Sobue or Hirohisa Watanabe

Brain and Mind Research Center

Nagoya University Graduate School of Medicine

65 Tsurumai-cho, Showa-ku, Nagoya, Aichi 466-8550 Japan

Tel.: +81-52-744-2385, Fax: +81-52-744-2384

E-mail: sobueg@med.nagoya-u.ac.jp (GS) or nabe@med.nagoya-u.ac.jp (HW)

Supplementary Information

Supplementary Table S1. Dual regression analysis results. List of clusters (size > 10) showing significant (FWE $p < 0.05$) relationship with age and sex for different resting state networks.

Contrast	Cluster #	No. of Voxels	T-stat	FWE p	Peak Location (MNI, mm)			Area/BA
					X	Y	Z	
**Dorsal default mode network**								
Age	1	178	3.75	0.019	14	-76	34	R Precuneus/BA7
	2	116	4.45	0.008	20	-8	-8	R Medial Globus Pallidus
nAge	1	631	4.87	0.001	6	-80	4	R Cuneus
**Primary visual network**								
Age	1	10333	5.32	0.001	-2	-34	-14	L Brainstem
	2	7454	5.46	0.000	-10	-52	30	L Precuneus
	3	1067	4.44	0.006	-12	30	18	L Anterior Cingulate
	4	399	3.65	0.022	32	56	18	R Superior Frontal Gyrus/BA10
	5	220	3.50	0.019	22	-100	-14	R Lingual Gyrus
	6	183	3.93	0.016	-18	14	-6	L Putamen
	7	86	3.87	0.024	-12	-12	44	L Cingulate Gyrus
	8	73	3.74	0.027	24	-36	12	R Extra-Nuclear
	9	71	2.83	0.045	-32	-82	-4	L Middle Occipital Gyrus
	10	70	3.21	0.034	-38	-52	16	L Superior Temporal Gyrus
	11	44	3.99	0.028	-8	-30	52	L Paracentral Lobule
	12	26	2.78	0.045	-28	-100	-16	L Inferior Occipital Gyrus
	13	16	2.69	0.048	24	58	2	R Superior Frontal Gyrus/BA10
nAge	1	324	3.80	0.010	-18	-76	-16	L Lingual Gyrus
**Left executive control network**								
Age	1	96	4.58	0.014	20	24	-4	R Caudate
**Right executive control network**								
Age	1	978	4.68	0.001	-18	14	14	L Caudate
	2	761	4.95	0.000	2	-34	-20	R Brainstem
	3	134	3.65	0.034	16	-36	62	R Postcentral Gyrus
	4	94	3.55	0.037	-18	-40	64	L Postcentral Gyrus
	5	73	4.04	0.021	22	-74	34	R Precuneus
	6	63	4.29	0.017	-28	-70	40	L Precuneus

	7	37	4.46	0.021	-34	-38	20	L Insula/BA13
nAge	1	650	4.33	0.004	54	-34	60	R Postcentral Gyrus
	2	84	3.96	0.018	32	2	42	R Middle Frontal Gyrus
**Anterior salience network**								
Age	1	549	4.88	0.001	-12	-28	-14	L Brainstem
	2	503	4.11	0.003	-52	14	-24	L Superior Temporal Gyrus
	3	131	5.14	0.003	-10	14	14	L Caudate
	4	125	3.84	0.014	46	-54	58	R Inferior Parietal Lobule/BA40
	5	125	4.67	0.006	-34	-46	42	L Inferior Parietal Lobule
	6	82	4.11	0.013	-14	-54	-14	L Cerebellum
	7	14	3.74	0.039	8	16	2	R Caudate
nAge	1	182	5.56	0.001	2	2	8	R Caudate
	2	127	4.44	0.009	58	14	-6	R Superior Temporal Gyrus
	3	46	5.15	0.006	0	-40	-6	L Cerebellum
	4	39	4.18	0.025	-2	-28	4	L Thalamus
**Visuospatial network**								
Age	1	1782	4.94	0.000	8	-4	48	R Cingulate Gyrus/BA24
	2	1195	4.41	0.002	-50	-50	48	L Inferior Parietal Lobule
	3	987	4.17	0.005	30	-8	6	R Putamen
	4	958	4.01	0.010	-60	-20	-20	L Inferior Temporal Gyrus
	5	946	3.79	0.006	18	-8	24	R Caudate
	6	681	4.53	0.003	52	-42	42	R Inferior Parietal Lobule
	7	604	4.09	0.006	-30	12	-30	L Superior Temporal Gyrus/BA38
	8	267	4.21	0.005	36	10	-24	R Superior Temporal Gyrus/BA38
	9	63	3.70	0.026	-32	-30	-26	L Parahippocampal Gyrus/BA36
	10	61	3.40	0.029	-18	-46	-6	L Parahippocampal Gyrus/BA19
	11	55	3.50	0.036	16	-16	76	R Superior Frontal Gyrus/BA6
	12	21	3.84	0.036	-22	30	42	L Middle Frontal Gyrus
	13	13	3.53	0.039	18	-18	-10	R Brainstem
nAge	1	72	3.87	0.028	-54	-26	58	L Postcentral Gyrus/BA1
Sex	1	86	4.61	0.015	10	-50	26	R Cingulate Gyrus
	2	45	4.43	0.024	-8	-52	30	L Precuneus
**High visual network**								

Age	1	7950	4.53	0.001	-2	-66	-44	L Cerebellum
	2	1651	4.32	0.003	62	-28	2	R Superior Temporal Gyrus/BA22
	3	978	3.91	0.005	-48	10	-2	L Insula
	4	951	4.32	0.001	44	-30	38	R Postcentral Gyrus
	5	268	5.30	0.001	-4	28	-6	L Anterior Cingulate
	6	149	3.53	0.018	52	12	18	R Inferior Frontal Gyrus
	7	111	3.99	0.018	-20	-4	-4	L Lateral Globus Pallidus
nAge	1	5143	4.47	0.000	-36	-48	-26	L Cerebellum
	2	422	3.98	0.006	40	-48	-26	R Cerebellum
	3	205	3.76	0.018	32	-66	-2	R Middle Occipital Gyrus
	4	112	3.42	0.026	2	-94	-14	R Lingual Gyrus
**Precuneus network**								
Age	1	438	4.71	0.001	20	-8	76	R Superior Frontal Gyrus
	2	352	4.28	0.005	66	-26	38	R Postcentral Gyrus/BA2
	3	143	4.03	0.015	-36	-50	-18	L Fusiform Gyrus/BA37
	4	42	3.75	0.029	60	-40	50	R Inferior Parietal Lobule
	5	40	3.93	0.027	8	-6	44	R Cingulate Gyrus
nAge	1	181	5.64	0.001	-20	60	14	L Superior Frontal Gyrus
	2	162	4.76	0.006	-58	-12	12	L Transverse Temporal Gyrus/BA42
	3	158	4.30	0.009	20	-66	52	R Precuneus
	4	44	3.92	0.031	-16	-72	52	L Precuneus
nSex	1	43	4.62	0.017	-36	-18	56	L Precentral Gyrus/BA4
**Language network**								
Age	1	3662	5.00	0.000	12	-26	12	R Thalamus
	2	1456	3.39	0.015	30	-30	52	R Precentral Gyrus
	3	989	3.75	0.007	-18	-82	-14	L Lingual Gyrus
	4	408	3.73	0.008	-26	-38	-22	L Cerebellum
	5	339	4.23	0.006	-48	42	10	L Inferior Frontal Gyrus/BA46
	6	114	5.06	0.005	-10	-70	40	L Precuneus
	7	95	3.75	0.019	4	-30	-26	R Brainstem
	8	50	3.59	0.033	30	-28	-18	R Parahippocampal Gyrus
	9	40	3.23	0.039	22	-88	-20	R Fusiform Gyrus/BA18
	10	37	3.81	0.028	-6	-44	-42	L Brainstem
	11	35	3.77	0.032	-18	8	-22	L Inferior Frontal Gyrus
	12	33	3.79	0.028	-60	10	22	L Inferior Frontal Gyrus/BA45
	13	10	4.59	0.029	-6	-64	-38	L Cerebellum

nAge	1	129	4.15	0.019	-50	-34	10	L Superior Temporal Gyrus
	2	34	3.54	0.039	72	-34	8	R Superior Temporal Gyrus
	3	22	3.54	0.040	72	-34	18	R Superior Temporal Gyrus/BA42
	4	16	3.73	0.044	46	-80	0	R Middle Occipital Gyrus
**Medial sensorimotor network**								
Age	1	1990	4.28	0.001	-48	14	-32	L Superior Temporal Gyrus/BA38
	2	1640	5.28	0.000	-48	-36	48	L Inferior Parietal Lobule
	3	1211	5.56	0.000	58	-24	46	R Postcentral gyrus/BA2
	4	850	4.40	0.001	22	-74	-16	R Lingual Gyrus/BA18
	5	705	4.75	0.004	6	20	-4	R Anterior Cingulate/BA25
	6	629	3.64	0.008	-24	-72	-18	L Cerebellum
	7	396	4.62	0.002	42	-48	-30	R Cerebellum
	8	373	4.46	0.003	50	18	-24	R Superior Temporal Gyrus
	9	216	3.95	0.012	0	-8	18	L Thalamus
	10	186	3.67	0.015	42	-72	0	R Middle Occipital Gyrus
	11	157	4.80	0.002	12	-42	-2	R Parahippocampal Gyrus
	12	72	4.57	0.013	54	20	42	R Middle Frontal Gyrus
	13	49	4.59	0.019	12	30	62	R Superior Frontal Gyrus/BA6
	14	41	3.58	0.033	70	-28	2	R Superior Temporal Gyrus
nAge	1	2021	4.92	0.001	-20	-74	32	L Cuneus
	2	623	4.63	0.001	34	58	16	R Middle Frontal Gyrus
	3	468	3.76	0.015	34	4	30	R Precentral Gyrus
	4	153	2.75	0.037	34	-22	72	R Precentral Gyrus
	5	131	4.21	0.016	40	-18	16	R Insula/BA13
**Cerebellum**								
Age	1	1058	3.77	0.009	-30	-76	-14	L Lingual Gyrus
	2	370	4.37	0.005	-56	22	0	L Inferior Frontal Gyrus/BA47
	3	244	3.89	0.005	0	-56	20	L Posterior Cingulate
	4	218	5.30	0.003	54	20	-12	R Superior Temporal Gyrus
	5	182	4.54	0.005	-36	-32	12	L Transverse Temporal Gyrus
	6	156	4.15	0.012	52	-2	-40	R Inferior Temporal Gyrus/BA20
	7	136	3.67	0.025	54	-16	-28	R Fusiform Gyrus
	8	132	4.34	0.016	32	-72	-4	R Fusiform Gyrus
	9	63	4.16	0.018	34	-18	-30	R Parahippocampal Gyrus
	10	34	3.16	0.043	24	-48	10	R Precuneus

	11	16	3.51	0.041	-50	34	12	L Inferior Frontal Gyrus
	12	12	3.91	0.043	-34	-20	-26	L Parahippocampal Gyrus
nAge	1	1559	4.89	0.000	4	-4	0	R Extra-Nuclear
	2	329	4.32	0.007	22	-24	-4	R Lateral Geniculum Body
	3	276	4.54	0.007	-6	-94	20	L Cuneus/BA19
	4	245	3.92	0.010	-26	-8	-4	L Putamen
	5	37	3.33	0.037	-30	-22	2	L Putamen
	6	27	3.55	0.033	-16	-28	-26	L Brainstem
	7	24	3.97	0.026	20	-24	-24	R Brainstem
**Lateral sensorimotor network**								
Age	1	624	4.38	0.001	-4	-74	2	L Lingual Gyrus/BA18
	2	504	4.87	0.001	-32	-50	34	L Supramarginal Gyrus
	3	243	4.07	0.010	-18	10	-10	L Putamen
	4	238	4.8	0.001	20	-46	76	R Postcentral Gyrus/BA7
	5	155	3.69	0.020	22	-70	40	R Precuneus
	6	139	4.96	0.005	28	-50	38	R Angular Gyrus
	7	88	3.78	0.021	64	-32	30	R Inferior Parietal Lobule/BA40
	8	56	3.64	0.025	-8	16	10	L Caudate
	9	56	3.98	0.024	-64	-44	-8	L Middle Temporal Gyrus
	10	55	4.42	0.018	-20	-16	24	L Caudate
	11	41	4.52	0.020	6	18	34	R Cingulate Gyrus
	12	27	4.73	0.020	-40	-80	-6	L Inferior Occipital Gyrus
nAge	1	155	5.35	0.001	2	0	12	R Extra-Nuclear
	2	40	4.88	0.015	26	-54	2	R Parahippocampal Gyrus/BA30
Sex	1	91	5.01	0.007	-6	30	-28	L Rectal Gyrus
**Ventral default mode network**								
Age	1	2604	4.85	0.001	-30	12	-24	L Superior Temporal Gyrus
	2	469	4.42	0.001	8	4	-4	R Extra-Nuclear
	3	169	4.01	0.008	-2	-12	14	L Thalamus
	4	70	3.84	0.029	-16	-74	2	L Lingual Gyrus
	5	47	4.43	0.019	50	6	-34	R Middle Temporal Gyrus
	6	46	5.79	0.004	14	-76	-38	R Cerebellum
	7	12	3.58	0.039	-32	-38	66	L Postcentral Gyrus
	8	12	4.09	0.034	-24	-52	64	L Superior Parietal Lobule/BA7
nAge	1	889	4.34	0.005	-2	-48	0	L Cerebellum
	2	630	4.18	0.004	24	-48	6	R Calcarine

	3	47	3.54	0.032	34	-34	-38	R Cerebellum
	4	22	3.81	0.043	-38	-92	16	L Middle Occipital Gyrus
Sex	1	30	5.00	0.020	-4	-62	34	L Precuneus

FWE – family-wise error; nAge – negative relationship with age; nSex – negative relationship with sex, BA – brodmann area; L – left; R – right

Supplementary Table S2. List of the 499 regions-of-interest used in whole brain network analysis.

ROI#	x	y	z	LOBE	RSN	SIZE	ANATOMICAL LABEL
1	6	-80	28	3	0	745	L/R cuneus/BA19
2	6	-94	-14	3	0	336	R lingual gyrus/BA17
3	26	-12	62	1	0	630	R middle frontal gyrus/BA6
4	-2	36	-18	1	0	834	L/R medial frontal gyrus/BA11
5	-22	-64	62	2	0	397	L superior parietal lobule/BA7
6	-30	-66	34	2	0	573	L precuneus
7	-10	-78	-46	6	0	188	L inferior semi-lunar lobule
8	-26	56	26	1	0	377	L middle frontal gyrus
9	64	-24	38	2	0	88	R postcentral gyrus/BA1
10	-22	-34	-22	6	0	511	L culmen
11	2	-10	50	5	0	796	L/R cingulate gyrus
12	-48	-36	22	2	0	902	L inferior parietal lobule
13	-62	-20	-20	4	0	518	L inferior temporal gyrus/BA20
14	-22	-68	6	5	0	567	L posterior cingulate/BA30
15	-28	-8	6	7	0	526	L lentiform nucleus/putamen
16	-4	-84	-2	3	0	539	L/R lingual gyrus
17	-20	54	-16	1	0	569	L superior frontal gyrus
18	-4	-32	12	7	0	561	L/R extra-nuclear
19	-42	-24	6	4	0	533	L superior temporal gyrus
20	2	-54	-46	6	0	537	L/R cerebellar tonsil
21	-38	-24	60	1	0	492	L precentral gyrus/BA4
22	44	-22	14	7	0	642	R insula/BA13
23	-12	-26	42	5	0	448	L cingulate gyrus/BA31
24	44	26	4	1	0	686	R inferior frontal gyrus
25	-20	-60	-46	6	0	594	L cerebellar tonsil
26	-38	4	34	1	0	363	L inferior frontal gyrus
27	8	-16	70	1	0	309	R medial frontal gyrus
28	-34	18	-16	1	0	498	L inferior frontal gyrus/BA47

29	28	-64	-40	6	0	479	R pyramis
30	4	-74	-36	6	0	389	L/R pyramis of vermis
31	50	-24	52	2	0	392	R postcentral gyrus
32	22	-86	32	3	0	566	R cuneus
33	-22	-38	68	2	0	511	L postcentral gyrus
34	4	2	-10	5	0	559	L/R anterior cingulate
35	-26	-50	4	5	0	228	L parahippocampal gyrus
36	-32	-52	-20	6	0	572	L culmen
37	2	64	-10	1	0	468	L/R superior frontal gyrus
38	36	-44	64	2	0	357	R postcentral gyrus/BA5
39	40	-80	20	4	0	499	R middle temporal gyrus
40	2	-24	-4	8	12	616	L/R thalamus
41	26	-38	66	2	0	528	R postcentral gyrus
42	-56	-54	-12	4	0	435	L inferior temporal gyrus
43	-42	-66	4	4	0	393	L middle temporal gyrus
44	52	-2	46	1	0	389	R middle frontal gyrus
45	-42	-50	-44	6	0	679	L cerebellar tonsil
46	-24	52	4	1	0	336	L superior frontal gyrus/BA10
47	2	-88	22	3	0	600	L/R cuneus
48	66	-44	2	4	0	252	R middle temporal gyrus
49	-58	-2	16	1	0	721	L precentral gyrus
50	-14	10	-20	1	0	362	L medial frontal gyrus
51	-62	-22	32	2	0	278	L postcentral gyrus
52	46	38	-14	1	0	369	R inferior frontal gyrus/BA47
53	14	34	28	5	0	331	R anterior cingulate
54	-14	-22	68	1	0	277	L precentral gyrus/BA6
55	46	46	-4	1	0	304	R middle frontal gyrus
56	-40	40	14	1	0	498	L middle frontal gyrus/BA10
57	44	8	44	1	0	399	R middle frontal gyrus
58	-6	-76	-18	6	0	404	L/R declive
59	10	18	-20	1	0	465	L/R medialfrontal gyrus
60	-26	-96	-10	3	0	842	L lingual gyrus/BA18
61	2	-54	-28	6	0	741	L/R culmen
62	-44	2	-12	1	0	589	L superior temporal gyrus
63	32	-38	-10	5	0	415	R parahippocampa gyrus/BA37
64	28	6	-40	4	0	1118	R sub-gyral
65	-28	-10	-28	5	0	654	L parahippocampa gyrus
66	14	54	4	1	0	602	R medial frontal gyrus/BA10
67	26	-66	8	5	0	347	R posterior cingulate

68	8	-40	56	1	0	405	R paracentral lobule
69	10	-86	-30	6	0	399	R declive
70	40	-18	66	1	0	171	R precentral gyrus
71	4	24	20	7	0	456	L/R extra-nuclear
72	-32	34	34	1	0	607	L superior frontal gyrus/BA9
73	52	-36	6	4	0	371	R superior temporal gyrus
74	4	-40	-2	5	0	499	L/R parahippocampal gyrus
75	-20	2	64	1	0	422	L middle frontal gyrus
76	48	-74	-18	4	0	264	R fusiform gyrus/BA19
77	46	40	14	1	0	342	R inferior frontal gyrus/BA46
78	-20	24	46	1	0	512	L middle frontal gyrus/BA8
79	-44	-70	26	4	0	404	L middle temporal gyrus
80	-48	36	-4	1	0	492	L middle frontal gyrus/BA47
81	30	-42	10	7	0	256	R extra-nuclear
82	-48	4	-24	4	0	871	L middle temporal gyrus/BA21
83	-38	8	50	1	0	417	L middle frontal gyrus
84	40	24	-14	1	0	550	R inferior frontal gyrus
85	36	-32	46	2	0	342	R sub-gyral
86	10	-60	-42	6	0	426	L/R cerebellar tonsil
87	56	20	20	1	0	352	R inferior frontal gyrus/BA45
88	36	-12	-36	5	0	369	R uncus/BA20
89	24	-54	-24	6	0	415	R culmen
90	6	-92	8	3	0	367	L/R cuneus
91	60	-20	-24	4	0	486	R inferior temporal gyrus
92	34	-8	50	1	0	357	R middle frontal gyrus
93	62	-52	38	2	0	79	R supramarginal gyrus
94	-18	-56	-16	6	0	264	L declive
95	4	-36	-16	8	0	218	R midbrain
96	32	-10	10	7	0	265	R claustrum
97	-40	-4	-46	4	0	401	L inferior temporal gyrus
98	-44	4	4	7	0	444	L insula/BA13
99	64	-8	32	1	0	350	R precentral gyrus/BA6
100	-46	32	28	1	0	347	L middle frontal gyrus
101	-26	-12	58	1	0	378	L middle frontal gyrus/BA6
102	66	-16	14	2	0	239	R postcentral gyrus/BA43
103	30	50	2	1	0	425	R middle frontal gyrus/BA10
104	14	-36	68	1	0	602	R precentral gyrus/BA4
105	-28	-84	20	3	0	242	L sub-gyral
106	26	38	-16	1	0	377	R middle frontal gyrus

107	-52	-28	-2	4	0	502	L superior temporal gyrus/BA22
108	28	64	-6	1	0	341	R superior frontal gyrus/BA10
109	24	6	62	1	0	192	R middle frontal gyrus
110	-22	-6	-20	5	0	545	L parahippocampa gyrus/Amygdala
111	22	46	38	1	0	279	R superior frontal gyrus/BA9
112	38	-54	62	2	0	116	R superior parietal lobule/BA7
113	-38	18	28	1	0	352	L sub-gyral
114	6	-92	-6	3	0	533	L/R lingual gyrus
115	-2	-12	-32	8	0	214	L pons
116	26	-82	-44	6	0	326	R pyramis
117	16	18	12	7	11	269	R caudate/caudate body
118	-2	-72	-26	6	0	567	L/R declive of vermis
119	2	8	36	5	0	544	L/R cingulate gyrus
120	26	-76	-34	6	0	443	R declive
121	24	14	-24	1	0	622	R inferior frontal gyrus/BA47
122	42	4	34	1	0	329	R inferior frontal gyrus
123	-18	8	-8	7	0	514	L lentiform nucleus/putamen
124	16	58	26	1	0	368	R superior frontal gyrus
125	-36	52	2	1	0	600	L middle frontal gyrus
126	26	-6	-6	7	0	684	R lentiform nucleus/putamen
127	2	30	-26	1	0	737	R rectal gyrus
128	54	-64	-12	3	0	325	R middle occipital gyrus/BA37
129	20	-62	26	3	0	287	R sub-gyral
130	12	68	8	1	0	362	R medial frontal gyrus/BA10
131	60	-30	10	4	0	655	R superior temporal gyrus
132	-2	-4	10	7	0	337	L/R extra-nuclear
133	-20	-68	44	2	0	350	L precuneus/BA7
134	2	18	-12	1	0	414	L/R subcallosal gyrus
135	18	26	56	1	0	392	R superior frontal gyrus
136	42	12	-30	4	0	699	R superior temporal gyrus
137	40	-90	8	3	0	214	R middle occipital gyrus/BA18
138	-42	-74	-32	6	0	356	L declive
139	48	-60	-30	6	0	338	R declive
140	46	-30	26	2	0	310	R inferior parietal lobule
141	-52	20	32	1	0	276	L middle frontal gyrus
142	2	-28	-26	8	0	820	L/R pons
143	-16	-90	6	3	0	310	L cuneus
144	46	-24	-26	4	0	517	R sub-gyral
145	28	-48	-34	6	0	412	R culmen

146	22	-72	58	2	0	249	R superior parietal lobule/BA7
147	46	-62	18	4	0	309	R superior temporal gyrus/BA39
148	-34	42	-16	1	0	466	L middle frontal gyrus
149	4	18	-4	5	0	533	L/R anterior cingulate/BA25
150	32	-68	-10	3	0	431	R sub-gyral
151	2	-26	62	1	0	425	R medial frontal gyrus
152	6	-54	72	2	0	171	R postcentral gyrus/BA7
153	2	-56	-6	6	0	135	L/R culmen
154	-2	-6	58	1	0	347	L/R medial frontal gyrus/BA6
155	24	-70	-48	6	0	273	R inferior semi-lunar lobule
156	30	-78	38	2	0	330	R precuneus/BA19
157	52	-10	-38	4	0	408	R inferior temporal gyrus/BA20
158	-8	16	6	7	0	230	L caudate/caudate head
159	-68	-32	8	4	0	322	L superior temporal gyrus
160	20	-56	4	5	0	479	R posterior cingulate
161	-54	-36	-22	4	0	642	L inferior temporal gyrus
162	-22	-74	26	3	0	400	L sub-gyral
163	-32	16	8	7	0	373	L insula
164	-60	-8	-14	4	0	476	L middle temporal gyrus
165	-36	-8	48	1	0	240	L middle frontal gyrus/BA6
166	2	-40	78	2	0	147	L/R postcentral gyrus
167	-16	-88	-20	3	0	274	L lingual gyrus
168	48	-56	30	4	0	274	R supramarginal gyrus
169	52	8	22	1	0	251	R inferior frontal gyrus/BA44
170	56	-18	28	2	0	270	R postcentral gyrus
171	2	-70	2	3	0	226	L/R lingual gyrus
172	-8	-96	20	3	0	346	L/R cuneus/BA19
173	34	8	54	1	0	328	R middle frontal gyrus
174	-6	-100	10	3	0	551	L/R middle occipital gyrus
175	-12	20	62	1	0	230	L superior frontal gyrus
176	8	-26	50	1	0	332	L/R paracentral lobule
177	-24	2	-40	5	0	458	L uncus/BA36
178	8	-28	-42	8	0	274	L/R pons
179	44	4	-14	4	0	636	R superior temporal gyrus
180	-4	-70	24	2	0	231	L precuneus
181	42	-2	-46	4	0	247	R middle temporal gyrus/BA38
182	-32	-86	-36	6	0	210	L uvula
183	52	8	-24	4	0	512	R superior temporal gyrus
184	36	-64	34	2	0	187	R angular gyrus/BA39

185	2	-18	-16	8	0	310	R substantia nigra
186	-52	-70	6	3	0	219	L middle occipital gyrus/BA19
187	32	-52	-12	3	0	324	R fusiform gyrus
188	22	-42	-46	6	0	164	R cerebellar tonsil
189	-36	-16	-34	5	0	283	L uncus/BA20
190	-22	26	-20	1	0	388	L middle frontal gyrus
191	-2	-62	10	5	0	121	L posterior cingulate/BA30
192	48	-56	-46	6	0	167	R cerebellar tonsil
193	-46	22	6	1	0	286	L inferior frontal gyrus
194	-38	-2	14	7	0	311	L insula/BA13
195	62	-38	28	2	0	254	R inferior parietal lobule
196	-50	-4	-36	4	0	544	L inferior temporal gyrus
197	22	-58	-48	6	0	289	R cerebellar tonsil
198	-24	-40	-44	6	0	296	L cerebellar tonsil
199	-36	12	-30	4	0	536	L superior temporal gyrus
200	64	-34	-14	4	0	292	R middle temporal gyrus
201	26	-12	-16	5	0	595	R parahippocampa gyrus
202	52	36	10	1	0	195	R inferior frontal gyrus
203	-44	-40	14	4	0	323	L superior temporal gyrus
204	56	-38	18	7	0	341	R insula/BA13
205	36	-46	-48	6	0	266	R cerebellar tonsil
206	0	-48	14	5	0	177	L/R posterior cingulate/BA29
207	-54	-42	52	2	0	69	L inferior parietal lobule/BA40
208	52	28	-2	1	0	285	R inferior frontal gyrus
209	-18	-76	-42	6	0	206	L pyramis
210	46	-14	-4	4	0	189	R superior temporal gyrus/BA22
211	-36	-24	42	1	0	179	L postcentral gyrus/BA3
212	2	30	-4	5	0	200	R anterior cingulate
213	-48	-82	4	3	0	296	L middle occipital gyrus
214	-26	64	-2	1	0	196	L superior frontal gyrus/BA10
215	4	-72	-46	6	0	169	R inferior semi-lunar lobule
216	-2	-2	74	1	0	53	L/R superior frontal gyrus/BA6
217	4	-88	38	3	0	58	L/R cuneus/BA19
218	28	-86	-34	6	0	245	R uvula
219	2	-2	30	5	0	324	L/R cingulate gyrus
220	4	-54	-38	6	0	430	L/R nodule
221	58	-16	-4	4	0	455	R middle temporal gyrus
222	54	-68	34	2	0	73	R angular gyrus
223	-34	-36	52	2	0	292	L postcentral gyrus

224	14	-78	-10	3	0	284	R lingual gyrus/BA18
225	6	-40	-34	8	0	185	L/R pons
226	54	-32	-28	4	0	266	R fusiform gyrus/BA20
227	-20	-58	-36	6	0	563	L cerebellar tonsil
228	40	16	12	7	0	370	R insula
229	-44	-10	10	7	0	447	L insula/BA13
230	-32	-74	-12	3	0	644	L lingual gyrus
231	22	-82	4	3	0	278	R cuneus
232	48	-56	10	4	0	193	R superior temporal gyrus/BA39
233	22	-76	40	2	0	257	R precuneus/BA7
234	56	-66	22	4	0	110	R middle temporal gyrus
235	-38	-48	-30	6	0	313	L culmen
236	60	-56	2	4	0	215	R middle temporal gyrus
237	4	-28	74	1	0	146	L/R paracentral lobule
238	32	32	30	1	0	182	R middle frontal gyrus
239	42	-54	-20	4	0	248	R fusiform gyrus
240	12	-52	40	2	0	252	R precuneus/BA7
241	24	-88	-20	3	0	172	R inferior occipital gyrus
242	46	-68	6	4	0	314	R middle temporal gyrus
243	28	-12	-26	5	0	691	R parahippocampal gyrus
244	20	-60	-16	6	0	319	R declive
245	-16	-50	66	2	0	358	L postcentral gyrus
246	6	-16	40	5	0	312	L/R cingulate gyrus
247	4	54	-22	1	0	550	L/R orbital gyrus
248	-44	-78	-18	3	0	207	L fusiform gyrus
249	48	-46	-22	4	0	273	R fusiform gyrus/BA37
250	20	-62	68	2	0	230	R superior parietal lobule/BA7
251	48	-36	44	2	0	163	R inferior parietal lobule
252	-60	-60	-2	4	0	217	L middle temporal gyrus
253	24	-54	58	2	0	496	R sub-gyral
254	8	-24	6	7	0	266	R thalamus/pulvinar
255	46	-70	-44	6	0	260	R pyramis
256	60	-8	-16	4	0	503	R middle temporal gyrus
257	-58	-12	32	1	0	617	L precentral gyrus/BA4
258	22	-66	44	2	0	315	R precuneus
259	60	-48	-10	4	0	276	R middle temporal gyrus
260	-2	-74	58	2	0	67	L/R precuneus
261	40	-8	18	7	0	186	R insula/BA13
262	-46	-60	-14	3	0	524	L sub-gyral

263	2	-40	8	7	0	42	L/R extra-nuclear
264	26	54	-16	1	0	396	R superior frontal gyrus
265	56	10	6	1	0	273	R precentral gyrus/BA44
266	-38	-74	16	4	0	209	L middle temporal gyrus
267	-4	-38	-40	8	0	588	L/R pons
268	-22	-82	42	2	0	252	L precuneus/BA7
269	-4	44	40	1	0	240	L/R medial frontal gyrus/BA6
270	-2	-18	16	7	0	293	L/R extra-nuclear
271	-10	-34	66	1	0	429	L paracentral lobule
272	12	46	46	1	0	233	R superior frontal gyrus
273	-34	26	2	1	0	445	L inferior frontal gyrus/BA47
274	68	-14	-10	4	0	75	R middle temporal gyrus/BA21
275	16	-36	44	5	0	246	R cingulate gyrus
276	58	-6	16	2	0	417	R precentral gyrus
277	2	-20	24	7	0	529	L/R extra-nuclear
278	36	44	34	1	0	199	R middle frontal gyrus/BA9
279	48	-58	-6	4	0	371	R inferior temporal gyrus
280	-40	-48	38	2	0	235	L supramarginal gyrus/BA40
281	42	-74	-30	6	0	325	R declive
282	24	-100	-8	3	0	269	R cuneus/BA18
283	-44	-56	10	4	0	295	L middle temporal gyrus/BA39
284	-2	-36	46	2	0	228	L/R precuneus
285	-16	-90	34	3	0	358	L cuneus/BA19
286	12	4	50	5	0	227	R cingulate gyrus/BA24
287	-30	34	-12	1	0	181	L middle frontal gyrus
288	26	60	20	1	0	324	R superior frontal gyrus/BA10
289	26	60	8	1	0	248	R superior frontal gyrus
290	-42	-2	54	1	0	236	L middle frontal gyrus/BA6
291	-46	-46	-22	4	0	190	L fusiform gyrus/BA37
292	38	-48	38	2	0	153	R supramarginal gyrus/BA40
293	10	20	64	1	0	133	R superior frontal gyrus/BA6
294	40	52	14	1	0	284	R middle frontal gyrus/BA10
295	56	16	36	1	0	33	R middle frontal gyrus/BA9
296	4	26	52	1	0	355	L/R medial frontal gyrus/BA8
297	34	-82	14	3	0	285	R middle occipital gyrus
298	2	-102	2	3	0	160	L/R cuneus
299	-12	8	20	7	0	282	L extra-nuclear
300	-32	-84	8	3	0	318	L middle occipital gyrus
301	-36	12	-42	4	0	300	L middle temporal gyrus

302	-12	-52	42	2	0	285	L precuneus/BA7
303	54	-8	30	1	0	396	R precentral gyrus
304	28	20	42	1	0	203	R middle frontal gyrus
305	-34	-28	-26	5	0	362	L parahippocampa gyrus
306	64	-26	-8	4	0	251	R middle temporal gyrus
307	2	-26	-14	8	0	778	L/R midbrain
308	-22	46	24	1	0	222	L superior frontal gyrus
309	-32	-36	-6	4	0	323	L sub-gyral
310	18	-2	24	7	0	231	R extra-nuclear
311	-2	34	54	1	0	264	L/R superior frontal gyrus/BA8
312	4	-20	-40	8	0	220	R pons
313	18	-68	-2	3	0	366	R lingual gyrus/BA19
314	30	-78	44	2	0	91	R superior parietal lobule
315	-48	-18	34	1	0	254	L postcentral gyrus
316	-32	4	-6	7	0	218	L claustrum
317	-60	-42	10	4	0	460	L superior temporal gyrus
318	4	-44	68	2	0	180	L/R postcentral gyrus
319	-54	-56	-26	4	0	235	L fusiform gyrus/BA37
320	-44	-20	20	7	0	310	L insula
321	18	-52	-8	5	0	404	R parahippocampa gyrus/BA19
322	34	-34	-22	4	0	465	R fusiform gyrus/BA20
323	-16	-12	68	1	0	359	L superior frontal gyrus/BA6
324	52	6	-38	4	0	262	R middle temporal gyrus
325	16	-2	68	1	0	321	R superior frontal gyrus
326	-20	-44	54	2	0	292	L paracentral lobule
327	38	-2	-2	7	0	343	R insula
328	28	-70	-26	6	0	256	R declive
329	58	-46	-20	4	0	340	R inferior temporal gyrus/BA37
330	-42	-40	62	2	0	394	L postcentral gyrus/BA2
331	42	-46	-38	6	0	516	R culmen
332	2	-36	38	5	0	430	L/R cingulate gyrus/BA31
333	26	12	-6	7	0	346	R extra-nuclear
334	58	-62	10	4	0	146	R middle temporal gyrus
335	-24	-60	-8	5	0	401	L parahippocampa gyrus
336	30	10	6	7	0	202	R extra-nuclear
337	-14	66	12	1	0	319	L superior frontal gyrus
338	-60	-48	-2	4	0	178	L middle temporal gyrus
339	-24	-12	-8	7	0	397	L lentiform nucleus/lateral globus pallidus

340	-36	-88	20	3	0	276	L superior occipital gyrus
341	4	-28	-34	8	0	374	L/R pons
342	-18	-50	-4	3	0	192	L lingual gyrus
343	36	42	18	1	0	235	R middle frontal gyrus
344	-64	-34	-8	4	0	360	L middle temporal gyrus
345	40	22	28	1	0	345	R sub-gyral
346	-36	-68	-26	6	0	382	L declive
347	-56	14	12	1	0	210	L inferior frontal gyrus/BA44
348	44	-2	10	7	0	240	R insula
349	-12	-70	-6	3	0	262	L lingual gyrus
350	10	-68	16	3	0	205	R precuneus/BA31
351	4	-38	-48	8	0	207	L/R medulla
352	-12	42	50	1	0	229	L superior frontal gyrus
353	-52	-18	-32	4	0	533	L fusiform gyrus
354	-18	-76	-14	3	0	222	L lingual gyrus
355	6	42	34	1	0	366	L/R medial frontal gyrus
356	-50	-30	54	2	0	324	L postcentral gyrus
357	-50	-72	16	4	0	248	L middle temporal gyrus/BA39
358	28	-72	-20	6	0	381	R declive
359	-50	-6	44	1	0	364	L precentral gyrus
360	-46	-34	16	4	2	16	L superior temporal gyrus
361	-58	-12	4	4	2	548	L superior temporal gyrus
362	-58	-20	14	2	2	390	L postcentral gyrus
363	56	-8	6	4	2	552	R precentral gyrus
364	22	6	2	7	3	42	R lentiform nucleus/putamen
365	12	-4	10	7	3	377	R thalamus/ventral anterior nucleus
366	-10	-6	8	7	3	264	L thalamus
367	-22	4	2	7	3	168	L lentiform nucleus/putamen
368	-46	22	26	1	3	23	L middle frontal gyrus
369	48	28	20	1	3	61	R inferior frontal gyrus
370	-6	-28	-40	8	3	25	L pons
371	-18	42	40	1	4	463	L superior frontal gyrus
372	-8	48	16	1	4	682	L/R medial frontal gyrus
373	-4	48	18	1	4	303	L/R medial frontal gyrus/BA9
374	-6	54	34	1	4	564	L/R superior frontal gyrus
375	-2	44	6	5	4	654	L/R anerior cingulate/BA32
376	-6	62	4	1	4	291	L/R medial frontal gyrus/BA10
377	4	48	-2	5	4	246	L/R anterior cingulate
378	2	38	18	5	4	405	L/R anterior cingulate

379	-14	60	20	1	4	238	L superior frontal gyrus
380	-4	42	-12	1	4	453	L/R medial frontal gyrus/BA11
381	-2	56	-10	1	4	497	L/R medial frontal gyrus
382	-48	-70	34	2	4	104	L angular gyrus
383	20	38	48	1	4	139	R superior frontal gyrus/BA8
384	-2	-58	24	5	4	722	L/R posterior cingulate/BA31
385	4	-60	34	2	4	316	L/R precuneus/BA7
386	-2	-48	30	5	4	440	L/R cingulate gyrus
387	2	-18	36	5	4	110	L/R cingulate gyrus
388	50	-66	32	2	4	35	R angular gyrus
389	-2	-12	4	7	4	126	L/R thalamus
390	-24	-30	-14	5	4	393	L parahippocampa gyrus
391	28	-24	-20	5	4	134	R parahippocampa gyrus
392	-32	-90	-2	3	5	846	L middle occipital gyrus/BA18
393	30	-88	-4	3	5	992	R middle occipital gyrus/BA18
394	42	-80	-8	3	5	451	R inferior occipital gyrus/BA19
395	24	-92	20	3	5	235	R cuneus
396	-50	26	-4	1	6	509	L inferior frontal gyrus
397	-54	-34	-6	4	6	341	L middle temporal gyrus
398	-56	-56	16	4	6	753	L superior temporal gyrus
399	-56	-50	32	2	6	187	L supramarginal gyrus
400	-52	-60	28	4	6	394	L superior temporal gyrus
401	48	28	-12	1	6	38	R inferior frontal gyrus
402	58	-52	18	4	6	716	R superior temporal gyrus
403	52	-32	-8	4	6	350	R middle temporal gyrus
404	-24	-82	-38	6	6	241	L tuber
405	-8	34	42	1	7	77	L medial frontal gyrus
406	-42	20	44	1	7	543	L middle frontal gyrus
407	-26	22	56	1	7	407	L superior frontal gyrus
408	-32	30	46	1	7	275	L middle frontal gyrus
409	-42	46	-4	1	7	367	L middle frontal gyrus
410	-44	-66	42	2	7	600	L inferior parietal lobule
411	-50	-52	50	2	7	415	L inferior parietal lobule/BA40
412	-34	-70	50	2	7	470	L superior parietal lobule/BA7
413	-60	-44	-14	4	7	363	L middle temporal gyrus
414	36	-70	-44	6	7	310	R pyramis
415	-24	-26	66	1	12	534	L precentral gyrus
416	-46	-18	48	1	12	460	L precentral gyrus/BA4
417	-36	-24	64	1	12	362	L precentral gyrus/BA4

418	38	-24	58	1	12	695	R precentral gyrus/BA4
419	50	-14	46	1	12	430	R precentral gyrus/BA4
420	24	-22	72	1	12	282	R precentral gyrus
421	2	-14	62	1	12	139	R medial frontal gyrus
422	2	-44	-26	6	12	630	L/R culmen
423	4	-60	-16	6	12	695	L/R declive
424	-2	-48	-12	6	12	230	L/R culmen
425	-18	-60	-26	6	12	438	L culmen
426	-42	36	30	1	8	89	L middle frontal gyrus
427	-60	-34	38	2	8	620	L inferior parietal lobule
428	-58	-44	38	2	8	341	L supramarginal gyrus/BA40
429	-8	-54	62	2	8	94	L precuneus
430	14	-30	44	5	8	44	R cingulate gyrus/BA31
431	28	-44	72	2	8	47	R postcentral gyrus/BA2
432	16	-54	68	2	8	81	R postcentral gyrus/BA7
433	58	-30	34	2	8	586	R inferior parietal lobule
434	60	-40	38	2	8	349	R inferior parietal lobule/BA40
435	-14	-24	6	7	8	76	L thalamus/pulvinar
436	-34	-44	-38	6	8	60	L culmen
437	-38	-16	-6	7	8	90	L insula
438	40	-8	-10	4	8	134	R sub-gyral
439	2	-28	28	5	9	424	L/R cingulate gyrus
440	4	-70	38	2	9	665	L/R precuneus/BA7
441	6	-74	48	2	9	386	L/R precuneus/BA7
442	-2	-80	38	2	9	293	L/R precuneus/BA7
443	-36	-58	42	2	9	106	L inferior parietal lobule/BA40
444	40	-64	46	2	9	99	R inferior parietal lobule
445	2	-76	12	3	10	994	L/R cuneus
446	-12	-60	6	5	10	115	L posterior cingulate
447	40	20	50	1	11	653	R middle frontal gyrus/BA8
448	46	30	30	1	11	564	R middle frontal gyrus
449	28	28	50	1	11	423	R middle frontal gyrus/BA8
450	36	32	42	1	11	234	R middle frontal gyrus
451	38	54	2	1	11	348	R middle frontal gyrus
452	48	-60	42	2	11	688	R inferior parietal lobule
453	40	-62	54	2	11	201	R inferior parietal lobule
454	50	-46	48	2	11	493	R inferior parietal lobule
455	6	38	46	1	11	61	R medial frontal gyrus/BA8
456	-42	-68	-42	6	11	505	L pyramis

457	-32	-74	-48	6	11	452	L inferior semi-lunar lobule
458	-30	-70	-34	6	11	687	L uvula
459	-14	-86	-32	6	11	370	L pyramis
460	-34	46	22	1	1	683	L middle frontal gyrus
461	-42	14	-4	1	1	237	L inferior frontal gyrus/BA47
462	-8	12	64	1	1	400	L superior frontal gyrus
463	-2	28	32	5	1	766	L/R cingulate gyrus/BA32
464	-2	4	64	1	1	180	L/R superior frontal gyrus/BA6
465	-2	20	46	5	1	218	L/R cingulate gyrus/BA32
466	-2	14	46	1	1	548	L/R cingulate gyrus/BA32
467	14	10	64	1	1	258	R superior frontal gyrus
468	28	46	28	1	1	454	R superior frontal gyrus
469	42	16	-2	7	1	296	R insula/BA13
470	-36	-58	-34	6	1	85	L culmen
471	36	-60	-32	6	1	134	R culmen
472	-14	-60	16	4	13	342	L sub-gyral
473	-26	10	52	1	13	246	L middle frontal gyrus
474	-30	-40	-16	4	13	117	L fusiform gyrus
475	-38	-82	32	3	13	381	L superior occipital gyrus/BA19
476	14	-54	12	5	13	435	R posterior cingulate
477	2	-50	54	2	13	490	L/R precuneus
478	8	-62	58	2	13	405	R precuneus
479	-10	-68	56	2	13	429	L superior parietal lobule/BA7
480	4	-58	46	2	13	319	L/R precuneus/BA7
481	26	32	38	1	13	130	R superior frontal gyrus
482	24	16	50	1	13	52	R middle frontal gyrus
483	30	-36	-20	4	13	87	R fusiform gyrus/BA20
484	44	-74	30	4	13	578	R angular gyrus
485	-28	-2	54	1	14	310	L middle frontal gyrus
486	-44	-36	46	2	14	709	L inferior parietal lobule
487	-34	-52	48	2	14	518	L inferior parietal lobule
488	-24	-68	46	2	14	277	L superior parietal lobule/BA7
489	-28	-56	58	2	14	220	L sub-gyral
490	-50	8	30	1	14	675	L inferior frontal gyrus
491	-48	24	18	1	14	359	L inferior frontal gyrus
492	-50	-68	-6	4	14	107	L inferior temporal gyrus
493	28	2	54	1	14	103	R middle frontal gyrus
494	32	-56	50	2	14	821	R superior parietal lobule/BA7
495	50	-28	44	2	14	309	R postcentral gyrus

496	48	12	28	1	14	294	R inferior frontal gyrus
497	50	-60	-12	4	14	64	R sub-gyral
498	22	-76	-50	6	14	42	R inferior semi-lunar lobule
499	34	-72	-28	6	14	60	R declive

Lobe: 1 – frontal, 2 – parietal, 3 – occipital, 4 – temporal, 5 – limbic, 6 – cerebellum, 7 – sub-lobar, 8 – brainstem; Resting state networks: 0 – other brain regions, 1 – salience, 2 – auditory, 3 – basal ganglia, 4 – dorsal default mode, 5 – high visual, 6 – language, 7 – left executive control, 8 – posterior salience, 9 – precuneus, 10 – primary visual, 11 – right executive control, 12 – sensorimotor, 13 – ventral default mode, 14 – visuospatial/dorsal attention; L – left; R – right; BA – Brodmann area
